# The Fission Yeast Cell Integrity Pathway: A Functional Hub for Cell Survival upon Stress and Beyond

**DOI:** 10.3390/jof8010032

**Published:** 2021-12-30

**Authors:** José Cansado, Teresa Soto, Alejandro Franco, Jero Vicente-Soler, Marisa Madrid

**Affiliations:** Yeast Physiology Group, Department of Genetics and Microbiology, Campus de Excelencia Internacional de Ambito Regional (CEIR)—Campus Mare Nostrum, Universidad de Murcia, 30071 Murcia, Spain; teresaso@um.es (T.S.); afranco@um.es (A.F.); jerovic@um.es (J.V.-S.)

**Keywords:** fission yeast, MAPK, Cell Integrity Pathway, stress

## Abstract

The survival of eukaryotic organisms during environmental changes is largely dependent on the adaptive responses elicited by signal transduction cascades, including those regulated by the Mitogen-Activated Protein Kinase (MAPK) pathways. The Cell Integrity Pathway (CIP), one of the three MAPK pathways found in the simple eukaryote fission of yeast *Schizosaccharomyces pombe*, shows strong homology with mammalian Extracellular signal-Regulated Kinases (ERKs). Remarkably, studies over the last few decades have gradually positioned the CIP as a multi-faceted pathway that impacts multiple functional aspects of the fission yeast life cycle during unperturbed growth and in response to stress. They include the control of mRNA-stability through RNA binding proteins, regulation of calcium homeostasis, and modulation of cell wall integrity and cytokinesis. Moreover, distinct evidence has disclosed the existence of sophisticated interplay between the CIP and other environmentally regulated pathways, including Stress-Activated MAP Kinase signaling (SAPK) and the Target of Rapamycin (TOR). In this review we present a current overview of the organization and underlying regulatory mechanisms of the CIP in *S. pombe*, describe its most prominent functions, and discuss possible targets of and roles for this pathway. The evolutionary conservation of CIP signaling in the dimorphic fission yeast *S. japonicus* will also be addressed.

## 1. Introduction

The ability to sense and respond to external and internal stimuli is critical for the survival of living organisms in an ever-changing environment. In eukaryotic cells, the highly conserved Mitogen Activated Protein Kinase (MAPK) pathways are specialized signal transduction cascades that detect environmental signals at the cell surface, which are transmitted to a set of downstream cytoplasmic and nuclear effectors to control multiple aspects of cell function, including metabolism, division, death, differentiation, and movement [1,2,3]. The organization of MAPK pathways is based in a modular structure that maintains a remarkable degree of evolutionary conservation [2,4,5]. The first module of the pathway includes the sensor/s and downstream effectors that recognize the stimulus, while the second transmission module comprises the three-tiered and highly conserved kinases known as the MAPK kinase kinase (MAPKKK), the MAPK kinase (MAPKK), and the MAP kinase (MAPK), which is the core component of the pathway. MAPKKKs are usually activated through phosphorylation in response to environmental changes by upstream signaling cascades composed by GTPases of the Rho or Ras families together with different kinases, although they can also become activated through oligomerization or changes in subcellular location. Once activated, MAPKKKs phosphorylate and activate the downstream MAPKK at the Thr and Ser residues. In turn, the MAPKK associates and dually phosphorylates the MAPK at two strongly conserved T and Y residues of a T-X-Y motif located within the activation loop [6,7]. Among the most biologically relevant downstream effectors phosphorylated by MAPKs are transcription factors, cell cycle regulators, chaperones, cytoskeletal proteins, RNA-binding proteins, and different cytoplasmic substrates [2,6,8].

The number of MAPKs varies depending on the eukaryotic organism. Mammalian cells carry five types of conventional MAPKs, which comprise the extracellular signal-regulated kinases Erk1 and Erk2, JUN amino-terminal kinase (JNK), p38, and Erk5. JNK and p38 are also known as “Stress-Activated Protein Kinases” (SAPK), because they become preferentially activated in response to multiple environmental cues and are involved in the regulation of apoptosis and stress responses [9]. On the other hand, MAPKs of the ERK group are activated in response to mitogenic signals elicited by growth factors and phorbol esters and regulate several aspects of cellular functions including proliferation, survival, growth, metabolism, migration, and differentiation in response to extracellular cues [10]. While JNKs have only been described in mammals, orthologs to ERK and p38 MAPKs are also present in yeasts, including the rod-shaped fission yeast *Schizosaccharomyces pombe*, which, during the last few decades, has been positioned as an excellent model organism to depict functional mechanisms of general significance in eukaryotes, particularly in the field of fungal biology. *S. pombe* has three MAPK-signaling cascades known as the mating-pheromone, the stress-activated (SAPK), and the cell integrity (CIP) pathways. The SAPK pathway is homologous to mammalian p38, while the CIP pathway shows strong homology with mammalian ERKs [11,12,13,14,15]. In this review we present an exhaustive and updated description of the organization, components, and multiple biological roles of the CIP in *S. pombe*, from its discovery almost 30 years ago to the present day.

## 2. Architecture and Organization of the Fission Yeast CIP

The cell wall (CW) is an essential structure for the viability of fungal cells. It is responsible for the maintenance of their morphology and provides protection against osmotic lysis and mechanical damage, as well as being a key target for various antifungals. The main components of the CW in *S. pombe* are polysaccharides, such as β-glucans (54–60%) and α-glucans (28–32%), which confer rigidity and are essential for cell integrity [16]. In this organism, the CIP regulates multiple processes such as CW synthesis and maintenance during stress, morphogenesis, cytokinesis, mRNA stabilization, vacuole fusion, and ionic homeostasis [15,17,18,19]. The core effector of the CIP is MAPK Pmk1, an extracellular signal-regulated kinase ERK ortholog, which becomes cyclically activated during cytokinesis [20] and also in response to multiple environmental changes including heat, osmotic and oxidative stresses, CW damage, and glucose depletion [15,18,21]. Accordingly, Pmk1-less mutants display cytokinetic defects as well as growth sensitivity to osmotic stress and CW-damaging agents. While Pmk1 activity is necessary to preserve cellular integrity under multiple circumstances, its constitutive hyperactivation can be harmful [15,17,18,19,22,23,24]. Pioneering studies demonstrated that the Ca^2+^/calmodulin-dependent protein phosphatase calcineurin (Ppb1) and Pmk1 pathway play antagonistic roles during chloride homeostasis [25]. Based on this negative interaction between calcineurin and Pmk1, a genetic screen was developed to isolate mutants that were *v*iable in the presence of *i*mmunosuppressant and *c*hloride ion (a phenotype called *vic*), which allowed the identification of key members of the CIP. Indeed, the *vic* phenotype is a characteristic feature of CIP null mutants, since the absence of Pmk1 activity suppresses the strong chloride sensitivity of *ppb1*Δ mutants or of wild-type cells subjected to pharmacological inhibition of calcineurin activity with the immunosuppressant FK506 [25,26] (further details on this issue will be described in the “*Calcium homeostasis*” section below).

**Figure 1:**
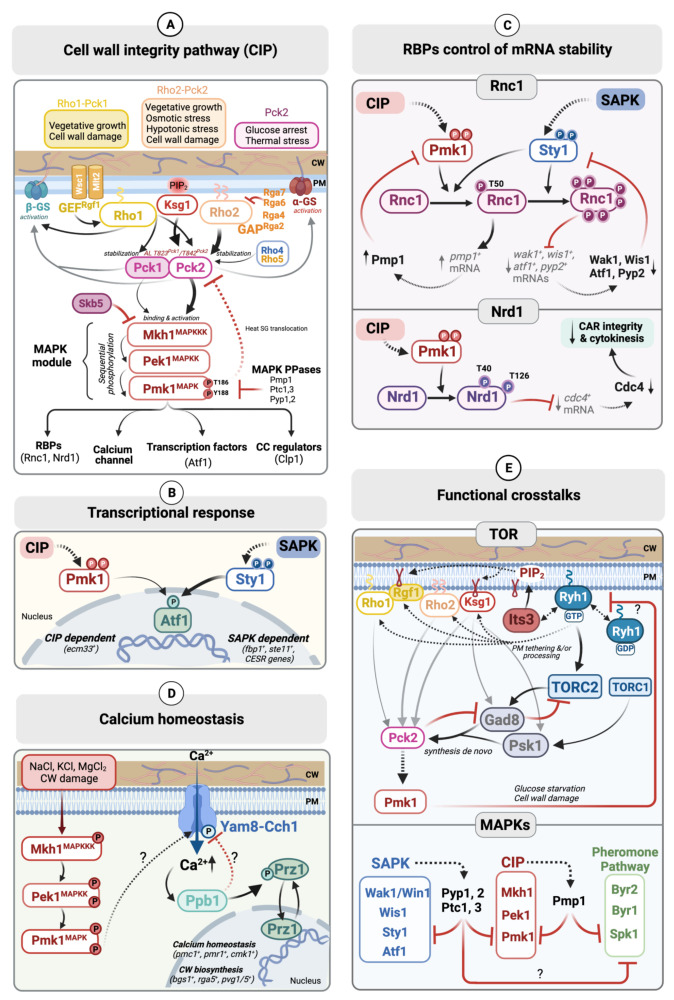
Organization and biological roles of the fission yeast Cell Integrity Pathway (CIP)**.** (**A**) The overall architecture and main components of the CIP in *S. pombe*. (**B**) Transcriptional regulation. Atf1, the main downstream transcription factor regulated by the stress activated MAPK pathway (SAPK) via Sty1 MAPK, can be also targeted by Pmk1 under specific stimuli, such as cell wall stress. The possibility that Pmk1 may, together with Sty1, regulate the expression of other Atf1-dependent genes besides *ecm33^+^* is currently unknown. (**C**) Control of mRNA-stability through RNA binding proteins (RBPs). The RBPs Nrd1 and Rnc1 become phosphorylated by Pmk1 during growth and stress to modulate the stability of mRNAs encoding the dual specificity phosphatase Pmp1 (Rnc1), and the myosin II essential light chain Cdc4 (Nrd1), thus allowing an accurate control of the Pmk1 activity threshold and cytokinesis, respectively. Sty1 also phosphorylates Rnc1 in vivo to prompt destabilization of mRNAs encoding SAPK components, resulting in reduced MAPK activity. (**D**) Regulation of calcium homeostasis. Pmk1 and calcineurin antagonistically regulate the activity of the Yam8-Cch1 channel complex to modulate the calcium influx in response to saline and cell wall stresses. (**E**) Functional cross-talks. Upper: Functional interaction between CIP and target of rapamycin (TOR) signaling pathways. Tor complex-2 (TORC2) and its main activator, the Rab GTPase Ryh1, positively control de novo synthesis of Pck2 and trigger Pmk1 activation in response to cell wall (CW) damage or upon glucose limitation. Ryh1 also promotes the plasma membrane targeting of several CIP upstream components and Pmk1 activation independently of TORC2 signaling. Conversely, activated Pmk1 down-regulates Ryh1 activity. Lower: The SAPK pathway negatively controls CIP signaling through the transcriptional induction of the MAPK phosphatases, Pyp1, Pyp2, Ptc1, and Ptc3, that dephosphorylate both Sty1 and Pmk1. Pmk1 dual specificity phosphatase Pmp1 also dephosphorylates Spk1, the MAPK of the pheromone response pathway. Dotted lines indicate that the molecular links and/or mechanisms have not been fully established. CW: cell wall; PM: plasma membrane; CC: cell cycle; RBP: RNA binding protein; and PPases: phosphatases. Please see the main text for specific nomenclature and details.

The main components of the CIP have been extensively characterized in *S. pombe* and will be described thereafter (Figure 1A). A short description of their biological roles and the corresponding *S. cerevisiae* orthologs are also shown in Table 1. Its overall architecture, as in every MAPK-signaling cascade, is based on a modular organization. The first and most upstream module comprises one or several sensors that specifically perceive the stimuli, plus a number of immediate downstream regulatory components. The second module comprises the signature MAPK transmission module, which is composed of three highly conserved kinases: MAPKKK, MAPKK, and MAPK. The nature of last module is heterogeneous depending on the signaling cascade and includes biologically distinct targets and effectors that become phosphorylated by the active MAPK (Figure 1A) [2,6,27]. Of note, very recently, we have characterized the main components of the CIP and their role during hyphal development in *S. japonicus*, a dimorphic fission yeast species. Our findings are presented in an accompanying research paper in this collection [28].

### 2.1. Upstream of the CIP MAPK Module

In *S. pombe*, the upstream elements of the CIP MAPK include, among others, several putative sensors, Rho-GTPases and their regulators (GEFs and GAPs), the phospholipid-dependent kinase (PDK), and two PKC orthologs (Figure 1A). Their structure, organization, and functional roles during CIP activation are described below.

#### 2.1.1. Sensors

Sensors are key components of cell integrity-signaling pathways, as they have the primary role for detecting changes in the CW and/or membrane structure and integrity in response to external stressors. In *S. cerevisiae*, as in other fungi, the transmembrane CW sensors belong to two different families, the WSC-type (Wsc1, Wsc2, and Wsc3) and MID-type (Mid2-Mtl1 pair) families, which function upstream and trigger CIP activation [29]. CW sensors show a cytoplasmic C-terminal tail that mediates downstream signaling, a transmembrane domain (TMD), and an N-terminal serine- and threonine-rich region (STR) that is highly mannosylated. In addition, the WSC-type sensors carry a cysteine-rich domain (CRD or WSC domain), while MID-type sensors have an N-glycosylated asparagine residue near the N-terminus, which mediates its interaction with CW polysaccharides [29,30]. Their characteristic conformation and ability to anchor both the plasma membrane and CW allow *S. cerevisiae* WSC and MID proteins to behave as mechanosensors that detect the perturbations at the CW and plasma membrane, which are transduced through their cytoplasmic tail to the CIP through Rom2 [29,31]. Rom2 is a Guanine Exchange Factor (GEF) that activates the GTPase Rho1, which in turn activates Pkc1, the only Protein Kinase C (PKC) ortholog present in budding yeast, which acts upstream of the CIP MAPK cascade [29,30,31].

In *S. pombe*, the two putative sensors Wsc1 and Mlt2 have been described as structural homologs of *S. cerevisiae* Wsc1 and Mlt1, respectively (Figure 1A) [32]. Wsc1 is located at active growth sites and the division septum, while Mlt2 is located at the cellular periphery. Mlt2 is necessary for fission yeast survival in response to different CW stresses, whereas Wsc1 overexpression activates CW biosynthesis [32]. Wsc1 and Mtl2 physically couple the CW with the plasma membrane and transmit signals mainly through Rgf1, a GEF that activates the essential GTPase Rho1, which in turn regulates β-(1,3)-glucan synthase activity and stabilizes the two PKC orthologs Pck1 and Pck2 [33]. Indeed, both Wsc1 and Mtl2 are necessary to maintain the physiological levels of active Rho1-GTP upon CW stress, and the overexpression of Rho1 or its GEFs suppresses the lethality caused by simultaneous deletion of *wsc1*^+^ and *mlt2*^+^ genes [32]. However, in contrast to the *S. cerevisiae* CWI sensors, CIP activity upon stress is not significantly compromised in the absence of Wsc1 and/or Mlt2, suggesting that they may regulate *S. pombe* CW integrity independently of this MAPK cascade [32]. Recent studies have proposed that both proteins perform CW mechanosensing activities that detect cell growth as a mechanical stress, i.e., as the thickness of the CW at the growing tips is very dynamic in fission yeast [34,35,36]. Wsc1 may represent an autonomous sensing module that detects mechanical stress at the CW and forms stable clusters that act as signaling platforms to recruit and activate downstream signaling elements to mechano-perception points [35,37]. This mechanosensing mechanism mediated by Wsc1 could represent a basal homeostatic module that, together with the CIP pathway, may contribute to fine-tuning of CW thickness and homeostasis [37].

#### 2.1.2. Rho-GTPases and Their Regulators

The Rho family GTPases are highly conserved and fundamental regulators in eukaryotes of multiple processes such as actin cytoskeleton dynamics, cell cycle, gene expression, vesicle trafficking, and cell polarity [38,39]. They perform their biological functions bound to biological membranes, for which they must be post-translationally modified [40]. Rho-GTPases oscillate between an active form bound to GTP that interacts with the downstream effectors, and an inactive form coupled with GDP. The exchange of GDP to GTP induced by Guanine Exchange Factors (GEFs) promotes their activation, while the hydrolysis of GTP to GDP is mediated by GTPase Activator Proteins (GAPs). Rho-GTPases are also negatively regulated by cytoplasmic Guanine nucleotide Dissociation Inhibitors (GDIs), which prompt GTPase removal from membranes [38]. *S. pombe* has six Rho GTPases (Rho1 to Rho5 and Cdc42), two of which, Rho1 and Cdc42, are essential [41,42]. Rho2 and, to a lesser extent, Rho1 are recognized as the key activating regulators of fission yeast CIP signaling (Figure 1A) [33].

Rho2 is a non-essential GTPase that localizes to the sites of active growth, and its deletion produces slightly rounded cells that are sensitive to glucanase treatment or staurosporine, a potent PKC inhibitor [6,43]. Fission yeast Rho2 regulates cell polarity, actin cytoskeleton organization, and CW biosynthesis by controlling the activity of α-glucan synthase Mok1 through the PKC orthologue Pck2 [6,44,45] (see below). In addition, Rho2 and Pck2 are the core upstream components that activate the CIP MAPK module (Figure 1A). Accordingly, fission yeast mutants lacking both proteins display a sharp *vic* phenotype [26]. Rho2 positively regulates the activation of the CIP MAPK module, fundamentally through Pck2, since the lethality associated with the Rho2 overexpression is totally suppressed in the absence of Pck2 or any of the MAPK module components [18,26]. Post-translational lipid modification of Rho2 mediates its proper membrane tethering and activation of the CIP [46,47,48]. This process takes place in vivo in three sequential steps. Rho2 becomes first farnesylated at C-197 by farnesyltransferase Cpp1 [26], and then the free carboxyl group of the isoprenylated cysteine is methylated by Mam4, a specific isoprenylcysteine-O-carboxylmethyltransferase (ICMT) [47]. Curiously, while Mam4 absence reduces Rho2-targeting to the plasma membrane, this effect is not observed with other methylated GTPases such as Rho1 or Cdc42 [47]. Finally, farnesylated and methylated Rho2 undergoes in vivo palmitoylation at C-196 by the DHHC palmitoyltransferase Erf2 [46]. This three-step lipidation process is essential for Rho2 full plasma membrane localization and the ensuing morphogenesis control and signaling to the CIP during unperturbed growth and stress [26,46,47,49].

The control of phosphoinositide turnover by eisosomes, which are multiprotein structures that generate linear invaginations at the plasma membrane of yeast cells, has also been proposed to modulate Rho2 localization and CIP activity [48]. This is based on the observation that Pmk1 activation during osmostress, which is Rho2-dependent, is impaired in a fission yeast hypomorphic mutant in the phosphatidylinositol (PI) 5-kinase Its3, and that this defect is suppressed in absence of eisosomes [48].

The four Rho2 GAPs identified to date, Rga2, Rga4, Rga6, and Rga7, have been shown to be involved in the control of CIP activity, since their single deletion prompts a significant, Rho2-dependent increase in Pmk1 MAPK activity during growth and stress (Figure 1A) [50,51,52]. The identity of the putative Rho2 GEFs, and their possible role during CIP signaling, is currently unknown. Rdi1, the only known Rho GDI in *S. pombe*, has been shown to negatively regulate the CIP, but it does so in a Rho2-independent fashion [46]. The fission yeast essential GTPase Rho1 is a functional homolog of human RhoA and *S. cerevisiae* Rho1. Rho1 localizes at active growth sites and is a direct activator of the (1, 3)-β-D-glucan synthase necessary for CW glucan synthesis and cytokinesis [41,44,53,54]. Other prominent effectors of Rho1 are the PKC orthologues Pck1 and Pck2 [53,55,56]. Subsequently, it was shown that Rho1 regulates CIP signaling in an additive and/or alternative way to Rho2 (Figure 1A) [21,57]. By using a mutant that expresses a hypomorphic allele of Rho1 (Rho1-596), it was demonstrated that Rho1 and Rho2 independently regulate Pck2 to maintain Pmk1 basal activity during vegetative growth [23,57]. According to the current model, Pck1, a second PKC ortholog, additionally regulates Pmk1 activity by acting as a specific target for Rho1, fundamentally in response to CW damage [23,57]. Rho1 GTPase activity is upregulated in vivo by three GEFs, Rgf1, Rgf2 and Rgf3, and is downregulated by three GAPs, Rga1, Rga5, and Rga8, together with Rdi1 GDI [58,59,60,61,62]. Rgf1 is the main GEF responsible for the maintenance of basal Rho1 GTPase activity in fission yeast cells during unperturbed growth, since its deletion strongly reduces the amount of the available Rho1-GTP pool. Activation of Rho1 by Rgf1 is necessary for the correct reorganization of actin cytoskeletons during the transition from monopolar to bipolar growth that occurs during *S. pombe* cell growth [63]. Later, Rgf1 was identified as a canonical member of the CIP, since its knockout blocked CIP activation upon different stresses, including osmotic stress. It was proposed that the Rgf1 positively regulates the CIP during this specific situation through the activation of Rho1 and Pck2 (Figure 1A) [21]. However, as described above, a Rho2–Pck2 branch positively regulates Pmk1 activation during vegetative growth and in response to many environmental cues including osmotic stress, which is totally Rho2-dependent [64]. It might be possible that Rgf1 could act as a Rho2 GEF under this specific situation, although this hypothesis has not been proven. On the other hand, the two redundant Rho1 GEFs, Rgf2 and Rgf3, do not seem to play a significant role in the regulation of CIP signaling, because Pmk1 activation upon stress was largely unaffected by their respective deletion [21].

Rho3, Rho4, and Rho5 GTPases have also been proposed as upstream regulators of the CIP besides Rho1 and Rho2, although their biological relevance is less clear. Rho3 has a general role in the secretion, exocytosis, and Golgi to endosome trafficking [38] and is a putative negative regulator of the CIP, as *rho3*Δ cells show a mild increase in Pmk1 phosphorylation and chloride sensitivity, which are typical features of CIP hyperactivation [46]. However, negative control of the CIP by Rho3 is likely an indirect effect and is independent of Rho2 function [46]. In fission yeast, Rho4 regulates cell morphology, septation and CW integrity [65,66,67], whereas Rho5 is a functional parologue of Rho1 (86% amino acids identity) that regulates cellular morphology and septation and also the survival of both vegetative cells and ascospores during the stationary phase [68,69]. Interestingly, *rho4*Δ and *rho5*Δ mutants display a *vic* phenotype and are growth-sensitive to micafungin, a specific inhibitor of CW beta-glucan synthase [70]. Pmk1 activation was lower in *rho4Δ* and *rho5Δ* mutants than in wild-type cells in response to heat shock, and their respective over-expression resulted in the stimulation of CIP signaling, mainly through Pck2 [70]. However, besides the plasma membrane, a notable portion of both GTPases localizes to internal membranes, suggesting that they may positively control the CIP through as yet unidentified target(s) [70]. In conclusion, Rho2, and to a lesser extent Rho1, are the main activating GTPases for the CIP (Figure 1A), while Rho3, Rho4, and/or Rho5 might contribute positively or negatively to modulate the activity of the pathway under very specific conditions.

#### 2.1.3. Phospholipid-Dependent Kinase-1 (PDK-1) and Protein Kinase C (PKC) Orthologs

The protein kinase C (PKC) family is a group of serine/threonine AGC kinases with essential roles in signaling pathways that participate in many different processes including cell proliferation, apoptosis, actin cytoskeleton remodeling, and ion channel modulation, among others [71]. All PKC isoforms share a basic structure that includes an N-terminal regulatory domain linked by a hinge region to a highly conserved C-terminal kinase domain [71]. The regulatory domain contains an autoinhibitory pseudosubstrate segment that is a key molecular switch during PCK activation, plus two putative membrane-binding modules named C1 and C2. The C1 module is conserved in all PKC isozymes, while the C2 module is found only in the conventional group of kinases [71]. The C-terminal kinase domain contains three conserved phosphorylation sites known as the activation loop (AL), turn motif (TM), and hydrophobic motif (HM), which are essential for catalytic activity [71,72,73,74]. *S. pombe* has two PKC orthologs known as Pck1 (111.78 kDa) and Pck2 (116 kDa), that share broad homology (70% identity in amino acid sequences within the catalytic domain), and have overlapping and essential roles in cell viability, since their simultaneous deletion is lethal [55,75]. Deletion of Pck1 does not cause obvious phenotypes, except for a moderate sensitivity to CW damage agents, and the appearance of a small percentage of cells showing mispositioned septa [55]. The *pck2Δ* mutants show clear defects in cell polarity and thin CWs and are hypersensitive to high temperatures or treatment with lytic enzymes [55,75]. While Pck1 overexpression prompts cellular elongation and a slight cell cycle delay, the overexpression of Pck2 is lethal and results in cells with very thick CWs and strong cytokinetic defects [55]. Both Pck1 and Pck2 contain all the typical domains of PKCs and, similar to the mammalian Rho family-responsive protein N kinases (PKNs), also show two polybasic coiled-coil HR1 motifs at their N-terminus that are responsible for binding to Rho1 and Rho2 GTPases [52,55]. Furthermore, both kinases are short half-life proteins that contain N-terminal PEST sequences (i.e., proline-, glutamic acid-, serine-, and threonine-rich sequences), which are involved in their degradation by the proteasome, and the interaction with activated Rho1 and Rho2 prompts their stabilization [52,55,74]. Pck1 and Pck2 co-localize with Rho1 and Rho2 in zones of polarized growth and relocate to the division zone before ring constriction [56,76,77]. Pck1 and Pck2 activation is controlled by several mechanisms, including autophosphorylation and phosphorylation by 3-phosphoinositide-dependent kinase-1 (PDK-1). Both modifications require prior binding and stabilization by Rho1 and/or Rho2 (see below) [55,74,78].

Rho1 and Rho2 regulate the biosynthesis of CW polymers (α- and β-D-glucan) through Pck1 and Pck2 and are also upstream activators of the CIP (Figure 1A) [18,26,46,52,55,57]. Rho1 regulates the synthesis of β-D-glucan, either directly or indirectly through Pck1 and Pck2 [55], while the synthesis of α-glucan is modulated by Rho2 strictly via Pck2 [45]. Moreover, the Rho2–Pck2 branch is the major upstream activator of the CIP upon stress, whereas both Rho1 and Rho2 target Pck2 to control basal Pmk1 activity during unperturbed vegetative growth [18,26,57]. It has been recently shown that Pmk1 promotes Pck2 translocation from the cell tips into stress granules upon heat stress, and this response that could contribute to prevent MAPK hyperactivation under such specific circumstances [79]. Instead, Pck1 plays a less meaningful role than Pck2 as a positive regulator of CIP, and its signaling activity seems biologically relevant only in response to CW damage by acting as a Rho1 effector (Figure 1A) [23,57].

PDK-1 is an essential AGC kinase family member that phosphorylates the AL of many AGC kinases including most PKC isozymes [71,80]. Ksg1 (65.66 kDa), one of the two *S. pombe* PDK-1 orthologs, plays an essential role in cell growth, conjugation, and sporulation [81,82]. It was initially shown that Ksg1 interacts with Pck1 and Pck2 in two hybrid assays [82]. Later, we demonstrated that Ksg1 is involved in CIP signaling through the activation of Pck1 and Pck2 [74,78]. In the current model, both Ksg1 and an autophosphorylation mechanism are responsible for the in vivo phosphorylation of Pck2 at the conserved T-842 within the AL during vegetative growth and in response to stress. AL phosphorylation, together with turn TM autophosphorylation at T-984 and binding to Rho1 and/or Rho2, prompt Pck2 stabilization and downstream activation of the CIP (Figure 1A) [74,78]. The equivalent AL phosphorylation site of Pck1, T-823, is also phosphorylated in vivo by Ksg1, and this modification is critical for Pck1 stabilization, catalytic activity, and biological function. Contrariwise, Pck2 is still stable and partially functional in the absence of AL phosphorylation [74,78]. Furthermore, the de novo synthesis of Pck1 and Pck2 is positively regulated by the TORC2 complex during vegetative growth and in response to several stresses (please see “*CIP and TOR signaling”*) [72,78]. Thus, despite their strong structural similarity and functional redundancy, the mechanisms that regulate the maturation, activation, and stabilization of Pck1 and Pck2 have a very different impact on their biological function as CIP activators [74]. In summary, a molecular network composed of Rho1, Rho2, Ksg1, Pck1, and Pck2 alternatively modulates CIP MAPK cascade signaling in *S. pombe* during vegetative growth and depending on the type of stress, as discussed below (Figure 1A) [57,64,83].

Recently, we have described that, in the dimorphic fission yeast *S. japonicus*, Pck1 and Pck2 are also key upstream regulators of the CIP pathway [28]. As in *S. pombe*, *S. japonicus* Pck2 is the major activator of Pmk1 during unperturbed growth. However, MAPK activation during CW damage, which is Pck1- and Pck2-co-dependent in *S. pombe*, is exclusively transmitted by Pck2 in *S. japonicus* [28]. Moreover, Pck1 and Pck2 antagonistically regulate hyphal differentiation through fine-tuning control of CIP activity. The putative conserved role of Rho1 and Rho2 GTPases and Ksg1 orthologs during Pck1/2 stabilization and activation is currently unknown [28].

### 2.2. The CIP MAPK Module

The core CIP MAPK module is composed of MAPKKK Mkh1, MAPKK Pek1, and MAPK Pmk1/Spm1 (Figure 1A). As with other MAPK module components, their structural integrity is essential for proper CIP signaling, and the absence of any of the three kinase components causes the typical features associated with loss of function of CIP signaling, including the *vic* phenotype, multiseptation, defective vacuole fusion, altered CW organization, and growth sensitivity to beta-glucan synthase inhibitors stress treatments [15,17,18,22,26].

#### 2.2.1. MAPKKK: Mkh1

Mkh1 (“Mek Kinase Homolog1”), is a 125.1 kDa protein, which was identified as the *S. pombe* CIP MAPKKK and is a structural homolog to the budding yeast CIP MAPKKK Bck1 [24]. Mkh1 associates in vivo with Pck1 and Pck2. However, while the Mkh1–Pck2 association is detected during vegetative growth and in response to stress, the Mkh1–Pck1 interaction has only been documented during CW stress [57]. This agrees with Pck2 being the major activator of CIP signaling during unperturbed growth. Once activated by Pck2, and occasionally by Pck1, Mkh1 transmits the activation signal to Pek1 and Pmk1, the remaining components of the MAPK module, through an in vivo interaction by forming a ternary complex (Figure 1A) [18,19,57]. Although Mkh1 is essentially a cytoplasmic protein, it can also be located at the septum area during cytokinesis and at the cell tips during the G1/S phase of the cell cycle [18,84]. Mkh1 targeting to the cell tips is regulated by Skb5 (Shk1 kinase binding protein) [84], an SH3 domain protein which is a direct activator of the p21-activated kinase (PAK) ortholog Pak1/Shk1 in fission yeast [85]. Skb5 inhibits Pmk1 MAPK signaling by downregulating Mkh1 localization to the cell tips, since its deletion results in the increased cell-tip accumulation of Mkh1 and the downstream activation of the CIP MAPK Pmk1 [84]. Similar to Mkh1, Pck2 is also tethered to the cell tips at the G1/S phase, suggesting that the Skb5–Mkh1 interaction may inhibit Pck2-dependent downstream signaling to the MAPK module [84].

#### 2.2.2. MAPKK: Pek1

Pek1 (“pombe mEK 1”), also named Skh1, is a 40.7 kDa protein encoded by the *pek1*^+^ gene, which shows a strong affinity with *S. cerevisiae* Mkk1 and Mkk2 MAPKKs [19,22]. Pek1, like Mkh1, is mostly a cytoplasmic protein that also localizes to the septum during cytokinesis [18]. Pek1 is phosphorylated and activated by Mkh1 in response to multiple stimuli (Figure 1A) [22]. Interestingly, in the absence of stress, inactive Pek1 binds to and inhibits Pmk1 activity. The dual activating/non-activating role of Pek1 allows this MAPKK to act as a molecular “all or nothing” switch during downstream signaling to Pmk1 MAPK depending on its phosphorylation status [22].

#### 2.2.3. MAPK: Pmk1

The MAPK Pmk1/Spm1 (“*S. pombe* MAP kinase 1”), a 48.26 kDa protein, was initially identified as a structural counterpart of Mpk1/Slt2 MAPK of *S. cerevisiae* and is ortologous to human ERK1/ERK2 and ERK5 [11,12,15,17]. Pmk1 is the core component of the CIP in *S. pombe* (Figure 1A), and its activation is fully dependent on the signaling transduced exclusively by Mkh1 and Pek1, which supports the fact that the CIP comprises an unbranched transmission MAPK module [17,18,24]. Similar to other MAPKs of the ERK-type, Pmk1 is activated by the upstream MAPKK (Pek1) through dual phosphorylation at T-186 and Y-188 located within the strongly conserved -TEY- activation motif [18,19,22]. In spite of these observations, it was later demonstrated that a threonine-monophosphorylated Pmk1 isoform performs, to a notable extent, many of the biological functions of the dually phosphorylated kinase [86]. On the contrary, tyrosine-monophosphorylated Pmk1 is not biologically active, despite showing an increased level of phosphorylation in this residue, which likely results from a trapping effect by Pek1 when the phosphorylation at the threonine residue is not available [86]. These observations support the fact that tyrosine phosphorylation is a prerequisite for the subsequent phosphorylation of Pmk1 at the threonine residue and that the dual phosphorylation of the MAPK is attained by an ordered and processive mechanism [86].

Although Pmk1 is constitutively localized in both the cytoplasm and the nucleus, it has also been detected in the mitotic spindle and in the septum during cytokinesis [18]. Contrary to mammalian ERK1/2, the sub-cellular targeting of Pmk1 is not significantly altered during stress or in the absence of the upstream module components Mkh1 or Pek1, suggesting that it becomes activated at the cytoplasm and/or septum, and that either the active or the inactive MAPK isoforms can freely translocate to the nucleus [18]. Detailed analysis of the biological consequences of the forced nuclear exclusion of Pmk1 has revealed that nuclear localization of the MAPK may be relevant for the full regulation of CW integrity at a transcriptional level. Conversely, many of the biological functions of Pmk1, including the regulation of chloride homeostasis and cellular separation, are executed by the cytoplasmic pool of the MAPK [87].

Pmk1 is cyclically activated during the unperturbed cell cycle, peaking at the G1-S transition during cytokinesis [20]. It also becomes activated in response to multiple environmental changes including heat, osmotic and oxidative stresses, CW damage, or glucose withdrawal [15,18,21]. Pmk1-less mutants display cytokinesis defects and are growth-sensitive to most of the above stresses, suggesting that CIP/Pmk1 activity is necessary to preserve cellular integrity in fission yeast under different biological scenarios [18,25,26,64,88]. Pmk1 activation must also be tightly controlled as its constitutive hyperactivation is detrimental, leading to notable morphological alterations that result in strong cell-growth defects [15,17,18,19,22,23,24].

The kinetics and magnitude of Pmk1 activation in response to environmental stimuli varies considerably depending on the stressor [18,64]. Some of the upstream regulatory components of the MAPK module may or may not function during signal transduction to the CIP according to the nature of the stimulus, suggesting the existence of alternative architectures for this signaling cascade [57,64]. For example, Pmk1 activation induced by hypertonic and hypotonic stresses is quick and transitory and relies completely on the Rho2–Pck2 branch, whereas MAPK activation induced during CW damage occurs progressively, reaches its maximum at long incubation times, and depends upon the activity of Rho1, Rho2, Pck1, and Pck2 [18,26,64,74]. Pmk1 activation in the absence of glucose occurs only after the total depletion of the sugar, requires de novo protein synthesis, is Rho2 independent, and involves Pck2 [83]. Contrariwise, Pck2 function is required to maintain a basal Pmk1 activity to allow further CIP activation during heat shock [64]. Rho4 and Rho5 might also play a positive role during this specific response [70].

*S. japonicus* Pmk1 shares a strong amino-acid sequence identity with *S. pombe* Pmk1 at the N- and C-lobes of the kinase domain, which includes the ERK-type TEY activation loop. However, a unique characteristic of the *S. japonicus* Pmk1 secondary structure is the absence of a 24 amino acids motif within the N-lobe, which includes the putative gatekeeper residue [28]. While *S. japonicus* Pmk1 also shows a nucleo-cytoplasmic location, it is not targeted to the septum during cytokinesis like its *S. pombe* counterpart. Despite these differences, comparative phenotypic analysis of *pmk1Δ* mutants revealed that regulation by the CIP of ionic homeostasis and CW integrity is conserved in both fission yeast species. Thereby, *S. japonicus* Pmk1 suppressed the phenotypes associated with a lack of CIP signaling when expressed in *S. pombe pmk1Δ* cells [28]. *S. japonicus* Pmk1 becomes activated by identical stressors to the *S. pombe* MAPK, except in response to glucose withdrawal, which does not occur in *S. japonicus*. Lack of MAPK activation upon this specific stimulus in *S. japonicus* might occur as the result of evolutionary loss, since this species utilizes glucose exclusively via fermentation and is respiration defective [28,89].

### 2.3. Downstream Targets of Pmk1

The main role of CIP is to ensure cellular adaptation in response to environmental changes through the phosphorylation of its targets. Only a few of direct Pmk1′s targets have been identified to date. The short list includes the transcription factor Atf1, the RNA binding proteins Rnc1 and Nrd1, and the cell cycle protein Clp1 (Figure 1A).

#### 2.3.1. Transcription Factor Atf1

The Atf-CREB family bZIP domain transcription factor Atf1 (59.71 kDa), is regarded as the key downstream effector of Sty1, which is the core MAPK of the SAPK pathway in *S. pombe*. Activated Sty1 associates with, phosphorylates, and stabilizes Atf1, which binds to promoters and ORFs of multiple stress-responsive genes, including the CESR (Core Environmental Stress Response), and modulates their expression in response to environmental cues [90,91,92,93,94,95]. However, some evidence supports the fact that Atf1 may also be targeted by Pmk1 under specific biological stimuli (Figure 1A). For instance, Pmk1 associates with and phosphorylates Atf1 specifically in response to CW damage, and it has been proposed that this transcription factor might enable the induced expression of genes required to maintain CW integrity [96]. One transcriptional target for the Pmk1–Atf1 branch is the *ecm33^+^* gene, encoding a glycosyl-phosphatidylinositol (GPI)-anchored cell surface protein that negatively modulates Pmk1 signaling [97] (Figure 1B). The *ecm33^+^* promoter shows a putative ATF/CREB-binding site, and its transcriptional induction also depends on Mbx1, a MADS-box transcription factor that controls gene expression during M-G1 cell cycle transition [98]. Mbx1 could also be a direct downstream target of Pmk1, but the in vivo association and phosphorylation by the MAPK have not been yet demonstrated. Aside from Ecm33, no additional CW gene target/s regulated via Pmk1–Atf1 transcription have been identified to date. As compared to wild-type cells, the growth sensitivity of cells lacking Atf1 to CW-damaging compounds (i.e., caspofungin) is rather modest. Moreover, a nuclear excluded version of Pmk1, which does not bind Atf1, is able to partially support growth in the presence of CW-damaging compounds [96]. Therefore, in contrast to *S. cerevisiae*, the biological significance of the transcriptional control of fission yeast CW integrity by the CIP seems rather modest.

Treatment of fission yeast cells with the pro-oxidant terc-buthyl hydroperoxide (TBH), induced the expression of a number of genes through Pmk1–Atf1 control in a Sty1-independent fashion [94], suggesting that this transcriptional branch is important for *S. pombe* survival in the presence of the oxidant. However, in strong contrast to the *pmk1Δ* mutant, *atf1Δ* cells are not growth-sensitive in the presence of TBH, supporting the fact that the CIP may promote fission yeast cellular adaptation to this stimulus more at a post-translational level [94]. Finally, it has been proposed that Pmk1 might work through Atf1 in addition to the canonical Sty1–Atf1 branch to promote the expression of genes involved in the adaptation of *S. pombe* from a fermentative to a respiratory metabolism such as fbp1+, encoding fructose-1,6-bisphosphatase [83]. Atf1 may thus act as a common transcriptional nexus between the SAPK and CIP MAPKs cascades under certain environmental scenarios.

#### 2.3.2. RNA Binding Proteins (RBPs) Nrd1 and Rnc1

RNA-binding proteins (RBPs) assemble into different mRNA-protein complexes and play key post-transcriptional roles in eukaryotes during splicing, mRNA transport, and the modulation of mRNA translation and decay, thus regulating essential cellular processes [99]. Phosphorylation of RBPs by MAPKs impacts their ability to bind and stabilize targeted mRNAs and influence cellular functions [100,101]. Indeed, Nrd1 and Rnc1, two of the ~140 RBPs present in the *S. pombe* proteome [102], have been described as direct downstream targets of Pmk1 during CIP signaling (Figure 1A). Their structural and functional characteristics, together with the biological significance of their functional control by Pmk1-dependent phosphorylation will be addressed exhaustively in a section below (“*Control of mRNA-stability through RBPs”*).

#### 2.3.3. Other Downstream Targets

The Cdc14-related serine/threonine protein phosphatase Clp1, also known as Flp1, regulates several aspects of mitotic exit in *S. pombe*. Clp1 locates at the nucleolus during vegetative growth and leaves this organelle to access its substrates when fission yeast cells face DNA damage agents or upon oxidative stress [103]. It has been shown that Pmk1 directly phosphorylates Clp1 at conserved -TP- MAPK phosphosites to promote its nucleoplasmic accumulation during genotoxic stress (Figure 1A). Accordingly, a Clp1 mutant lacking TP phosphosites showed a delayed nucleolus release during H_2_O_2_ treatment, whereas maintained phosphorylation at these sites prompted constitutive nucleoplasm targeting [104].

The plasma membrane Cch1–Yam8 channel complex that positively regulates the Ca^+2^ influx when *S. pombe* cells are subjected to salt stress, has been proposed as a direct phosphorylated substrate of Pmk1. However, the possibility that this is indeed the case remains to be confirmed. The antagonistic role in the fission yeast of calcineurin and CIP signaling during the control of Cch1–Yam8 activity and calcium homeostasis is addressed below (“*Calcium homeostasis*”).

### 2.4. Downregulation of Pmk1 Signaling by MAPK Phosphatases

Besides its activation, a tight control of Pmk1′s deactivation status is critical for the precise control of CIP signaling during unperturbed growth and environmental stresses. The primary negative regulator of CIPs during vegetative growth is Pmp1, a dual-specificity MAPK phosphatase that associates in vivo to Pmk1 and dephosphorylates the T-186 and Y-188 residues within the conserved -TEY- activation motif (Figure 1A) [25]. Accordingly, Pmp1 overexpression results in a strong *vic* phenotype in the absence of the calcineurin function, whereas its deletion increases Pmk1 dual phosphorylation in vivo [25]. The stability of *pmp1*^+^ mRNA is positively regulated by the RBP Rnc1, which is, in turn, phosphorylated and activated by Pmk1 to settle a negative feedback loop of MAPK signaling [105] (see “*Control of mRNA-stability through RBPs”* below). Later work demonstrated that the tyrosine phosphatases Pyp1 and Pyp2 and the threonine phosphatases Ptc1 and Ptc3, whose expression is mostly regulated by the SAPK pathway through Sty1 MAPK and the Atf1 transcription factor, also bind and dephosphorylate Pmk1 in vivo (Figure 1A). While Pyp1 and Ptc1 dephosphorylate Pmk1 during vegetative growth together with Pmp1, Pyp2 is only involved in MAP deactivation during stress [20]. Thus, SAPK-regulated MAPK phosphatases are involved in a crosstalk mechanism between the SAPK and CIP pathways (see *“Interplay with other fission yeast MAPK signaling cascades”* for specific details).

## 3. Main Regulatory Functions of the CIP

### 3.1. Control of Mrna-Stability through Rbps

#### 3.1.1. Nrd1

Nrd1 (Negative regulator of differentiation-1), also named Msa2 (Multicopy suppressor of sporulation abnormal mutant 2), is a 529 aa (57.76 kDa) RBP which was originally identified by its ability to repress sexual differentiation in *S. pombe* [106,107,108]. Conversely, deletion of the *nrd1^+^* gene causes fission yeast cells to initiate sexual development in the absence of nutrient starvation [106]. Further analysis revealed that Nrd1 blocks the onset of sexual development until cells reach a critical level of starvation by repressing the expression of genes induced by the transcription factor Ste11, which is a master transcriptional regulator of early sexual differentiation in fission yeast [106,109]. Nrd1 contains four copies of a putative RNA-recognition motif (RRM) distributed along its amino-acid sequence and was initially found to bind poly(U)-rich sequences with high affinity [106]. However, it is currently unknown if Nrd1 binds in vivo to mRNAs of either Ste11 or Ste11-dependent genes.

Later work identified Nrd1 as a multicopy suppressor of the thermo-sensitive and multiseptation phenotypes of cells expressing a hypomorphic version of the myosin II essential light chain Cdc4 (*cdc4-8* allele) [110]. Cdc4 plays an essential function to regulate fission yeast myosin II activity for the assembly and constriction of a contractile actin- and myosin-based ring (CAR) during cytokinesis, which enables the physical separation of daughter cells once mitosis has been completed [111]. While *cdc4^+^* mRNA and protein levels decrease upon Nrd1 deletion, both increase in response to Nrd1 overexpression. Furthermore, Nrd1 binds and stabilizes *cdc4^+^* mRNA in vivo [110]. This mechanism is exerted at least in part through its association with two UCUU motifs present in *cdc4^+^* mRNA, since their mutation results in a significant reduction in Nrd1′s binding affinity and lack of suppression of both the thermo-sensitive and multiseptation defects elicited upon Nrd1 overexpression [110]. Furthermore, Nrd1 is a direct target of Pmk1 MAPK, and Pmk1-dependent phosphorylation negatively regulates its ability to bind and stabilize *cdc4^+^* mRNA (Figure 1C). Pmk1 phosphorylates Nrd1 in vivo in at least at two threonine residues, T-40 and T-126, which lie at a two “perfect” MAPK phosphorylation sites (consensus sequence -PNTP-). These residues are located at the intrinsically disordered N-terminus and the RNA recognition motif-1, respectively [110,112]. The Pmk1 phosphorylation of Nrd1 at T-40 and T-126 is regulated in a cell cycle-dependent manner, being low at the M and G1-S phases and increasing sharply during G2. Conversely, *cdc4^+^* mRNA levels are maximal during the M to G1-S phases and strongly decrease during the G2 phase [110]. Taken together, these observations draw a scenario where Nrd1 binding and stabilization of the mRNA encoding the myosin II essential light chain are controlled by Pmk1-dependent phosphorylation during the cell cycle, thus promoting an accurate regulation of myosin-II function for CAR assembly and constriction during cytokinesis (Figure 1C).

Nrd1 localizes at the cytoplasm during unperturbed growth and is recruited to stress granules (SGs) in response to various stressful situations including heat shock, arsenite treatment, oxidative stress, and glucose starvation. This is accompanied by enhanced phosphorylation at MAPK phospho-sites T-40 and T-126 [113,114]. Accordingly, as compared to an unphosphorylatable Nrd1 mutant (Nrd1-T40A T126A), a phosphorylation-mimicked version of the RBP (Nrd1-T40D T126D mutant) translocates to SGs more quickly in response to stress, suggesting that MAPK-dependent phosphorylation at both residues promotes its recruitment to SGs [113]. Phenotypically, Nrd1 deletion induced resistance to sustained stresses and enhanced sensitivity to transient stresses [113]. However, the observation that the Nrd1 localization dynamics at SGs in response to stress do not change significantly in cells lacking Pmk1 [113] suggests that other MAPKs besides Pmk1 might regulate Nrd1 phosphorylation and subcellular localization at SGs. In support of this hypothesis, Nrd1 becomes phosphorylated in response to nitrogen starvation by Spk1, the MAPK of the pheromone-signaling pathway, and phosphorylation at T-126 plays an important role during the negative control of mating efficiency [114].

It has been proposed that Nrd1 adopts a beads-on-a-string structure, where each of its four RRM motifs, which are connected by flexible hinges, may undergo phosphorylation-induced conformational changes through these linkers [112]. Besides T-40 and T-126, the Nrd1 amino acid sequence has seven additional putative MAPK phosphorylation sites, three of which (S-5, S-93, and S-309) have been shown to become phosphorylated in vivo in global phosphoproteomic studies [115,116,117]. Altogether, this evidence suggests that alternative Nrd1 phosphorylation at multiple residues by the three *S. pombe* MAPK cascades might allow for the exquisite regulation of its biological functions as a RBP during unperturbed growth and in response to specific environmental cues. This control might somehow be exerted through its interaction with Cpc2, the ortholog of the receptor of activated C kinase (RACK1), that, in fission yeast, controls the extent of the activation of MAPK cascades from the ribosome, the cellular defense against oxidative stress, and the progression of the cell cycle [118]. Cpc2 associates with Nrd1 in vivo [108,113,114], and its deletion affects the formation of Nrd1 SGs upon specific stresses [113].

#### 3.1.2. Rnc1

Rnc1 (RNA-binding protein that suppresses calcineurin deletion 1), a 398 aa (43.38 kDa) RBP, was isolated in a genetic screen for regulators of cell integrity signaling based on the functional interaction between calcineurin and Pmk1. Rnc1 overexpression suppressed the strong chloride sensitivity of fission yeast cells lacking calcineurin (Ppb1) by repressing Pmk1 activity [105]. The Rnc1 amino-acid sequence contains three K-homology (KH) domains, which are also found as multiple copies in many eukaryotic RBPs that coordinate the different steps of RNA synthesis, metabolism, and localization [119]. Further analysis identified the mRNA encoding Pmp1, the dual specificity phosphatase that dephosphorylates Pmk1 during vegetative growth [25], as a direct target of Rnc1 (Figure 1C) [105]. Rnc1 directly binds *pmp1^+^* mRNA in vivo and in vitro through its three KH domains. Accordingly, the respective mutants in each of the conserved GXXG loops that are essential for RNA recognition and docking in KH-containing RBPs [120] failed to suppress the chloride sensitivity of calcineurin deletion [105]. Rnc1 stabilizes *pmp1^+^* mRNA by binding to several UCAU repeats located at its 3′ UTR [105]. This control is biologically relevant, since *pmp1^+^* mRNA levels decrease upon Rnc1 deletion, resulting in the increased basal phosphorylation of Pmk1 MAPK, whereas Rnc1 overexpression strongly reduces MAPK activity (Figure 1C) [105]. Importantly, activated Pmk1 binds and phosphorylates Rnc1 in vivo at a MAPK consensus phospho-site located at a threonine residue at position 50, and this post-translational modification enhances the Rnc1 binding and stabilization of *pmp1^+^* mRNA, thus posing Rnc1 as key component of a negative feedback loop of cell integrity MAPK signaling (Figure 1C) [105,121]. In agreement with this model, expression of an unphosphorylatable Rnc1 mutant at T-50 (T50A) failed to suppress the chloride sensitivity of *ppb1Δ* cells, whereas phosphorylation-mimic mutants (T50D or T50E), were more potent suppressors of this phenotype than the wild-type RBP [105,121].

Further studies revealed that Rnc1 localization at the cytoplasm is mediated by a putative Nuclear Export Signal (NES), present at the N-terminus, that mediates its nuclear to cytoplasm shuttling. Curiously, this mechanism is not mediated by the exportin-1 ortholog Crm1 and relies on unknown exportin(s) [122,123]. Rnc1 nucleo-cytoplasmic export shuttling is also exerted by the nucleoporin RNA export factor Rae1 [122,123]. An Rnc1 mutant version lacking NES accumulates in the nucleus and fails to bind *pmp1^+^* mRNA, thus prompting its destabilization and the enhancement of Pmk1 activity. Similarly, Rnc1 mutants at the KH domains do not bind *pmp1^+^* mRNA and accumulate in the nucleus due to the loss of nuclear exports of the mRNA-protein complex. Thus, nuclear export of Rnc1 requires an mRNA-binding ability and the mRNA export factor Rae1 [122,123].

Similar to Nrd1, Rnc1 is a component of the SGs that relocates to these structures as a result of different stimuli [124]. Rnc1 deletion results in decreased SGs, whereas Rnc1 overproduction induced a strong accumulation and aggregation of SGs even in the absence of stress, indicating that it plays a positive role in their formation. Rnc1 prompts SG assembly in response to stress dependently or independently of its RNA-binding activity, suggesting that different signaling pathways may target Rnc1 during the regulation of this process [124].

Recent work has shown that, besides Pmk1, the MAPK of the SAPK pathway Sty1 phosphorylates Rnc1 in vivo at multiple phosphosites (T-45, T-50, T-171, T-177, S-278, and S-286) during vegetative growth and stress (Figure 1C). These modifications trigger Rnc1 binding through its KH domains and destabilization of mRNA targets encoding both the activators (Wak1 MAPKKK and Wis1 MAPKK) and downregulators (Atf1 transcription factor and Pyp1 and Pyp2 phosphatases) of Sty1 phosphorylation (Figure 1C) [125]. Biologically, this control results in an overall reduction of Sty1 activity through a negative feedback mechanism via Rnc1 that ensures a precise control of fission yeast cell-size progression during vegetative growth and in response to acute stress [125]. Accordingly, cells expressing a mutated version of the RBP at the KH-domains, which are unable to bind mRNAs (Rnc1-m3KH) or lack phosphorylatable MAPK sites (Rnc1-S/T6A), phenocopy *rnc1Δ* cells and show increased Sty1 activity, reduced cell length at division, and enhanced tolerance to heat shock [125].

Altogether, this evidence presents an intriguing situation, where alternative phosphorylation of Rnc1 by Pmk1 and Sty1 MAPKs may prompt either mRNA stabilization (Pmp1 mRNA) or destabilization (Wak1, Wis1, Atf1, and Pyp1 and Pyp2 mRNAs), during unperturbed growth and stress (Figure 1C). The differences in Rnc1 binding affinity towards the target mRNAs might rely upon strong conformational changes induced by the alternative phosphorylation of MAPK phospho-sites, which are located outside of the KH domains and lie within intrinsically disordered regions of the protein [125]. The shared modulation of Rnc1 mRNA binding ability by the cell integrity and stress-activated MAP kinases Pmk1 and Sty1 is, to a certain point, an elegant example of MAPK crosstalk that allows for precise self-regulation during growth and in response to environmental changes.

### 3.2. Calcium Homeostasis

The first evidence suggesting that CIP signaling is involved in the regulation of ionic homeostasis in fission yeast came from the observation that mutants lacking Pmk1 exhibit differential growth sensitivity in the presence of cations. Indeed, as compared to wild-type cells, *pmk1Δ* cells are tolerant to sodium chloride, whereas they are sensitive to the presence of potassium and calcium salts in the growth media [17,18]. Later work established a putative functional link during the control of ionic homeostasis between the CIP and the Ca^2+^/calmodulin-dependent protein phosphatase Calcineurin (Ppb1). The strong chloride sensitivity of fission yeast cells lacking calcineurin activity (i.e., in *ppb1Δ* cells or through specific pharmacological inhibition with Tracolimus/FK506) could be totally abrogated by the simultaneous deletion of Pmk1. This phenotype, named *vic* (*v*iable in the presence of *i*mmunosuppressant and *c*hloride ions), set the experimental ground for the identification of key core components of the CIP such as Pmp1, Pek1, and Rnc1 [22,25,105] and for the precise assessment of the biological relevance of upstream regulators of the MAPK module including Rho1, Rho2, Pck1, and Pck2 [57,64]. The *vic* phenotype is also present in the lack of function mutants in CIP signaling in *S. japonicus*, thus supporting its conservation within the *Schizosaccharomyces* genus [28].

Calcium homeostasis is critical for the regulation of multiple physiological processes in eukaryotes. Accordingly, minimal changes in its cytoplasmic levels lead to the activation of several calcium-sensing proteins, including calcineurin, which results in the induction of various downstream transduction pathways [126]. In *S. pombe,* activation of the calcineurin catalytic subunit Ppb1 in response to calcium accumulation triggers the dephosphorylation and nuclear translocation of the nuclear factor Prz1, an activated T-cell family (NFAT) transcription factor that regulates the expression of genes showing Calcium Dependent Response Element (CDRE)-like motifs at their regulatory sequences [126,127,128]. Prz1 regulates the expression of genes involved in calcium homeostasis, such as *pmc1^+^, pmr1^+^, ncs1^+^, cmk1^+^*, and *prz1^+^* [126,127,128,129,130], and also CW biosynthesis genes such as *bgs1^+^, rga5^+^, omh1^+^, pvg1^+^*, and *pvg5^+^* [131]. While mutants lacking Prz1 are hypersensitive to calcium, *ppb1Δ* cells exhibit additional phenotypes including cytokinesis and cell polarity defects [126,132]. Importantly, the overexpression of Pmp1 phosphatase, which negatively regulates Pmk1 activity, fully suppressed the chloride hypersensitivity, but not the calcium hypersensitivity, of *ppb1Δ* cells. From these observations, it was hypothesized that Prz1 is not the sole target of calcineurin during the control of ionic homeostasis, and that this control is exerted through at least two different signaling pathways instead. One requires Prz1-dependent transcriptional regulation that regulates calcium homeostasis, whereas a second and unknown mechanism would function antagonistically with the CIP to modulate cellular morphogenesis and chloride homeostasis [126].

The transient receptor potential (TRP) calcium channels, Trp1322 and Pkd2, play significant functional roles during fission yeast-CW biosynthesis and membrane trafficking [23,133]. Similarly, the Yam8 channel (also known as Ehs1), has also been involved in CW integrity control and calcium import [134]. By performing live measurements of cellular cytoplasmic Ca^2+^ levels, it was found that Trp1322 and Pkd2 directly regulate calcium influx in *S. pombe*, whereas exposure of fission yeast cells to KCl, NaCl, and MgCl_2_, elicits calcium import and cytoplasmic accumulation through the Yam8/Cch1 channel complex, thus resulting in calcineurin activation [127,135]. Those treatments prompted a further increase in cytoplasmic calcium content in either *ppb1Δ* cells or FK506-treated cells as compared to wild-type cells, and this feature was suppressed in the absence of Yam8 and/or Cch1. Therefore, calcineurin may directly downregulate the Ca^2+^ influx activity of the Yam8–Cch1 channel complex [135].

Remarkably, fission yeast mutants lacking either Pmk1 or upstream CIP elements exhibit extremely low cytoplasmic calcium levels during unperturbed growth, and their deletion suppresses the synergistic increase in calcium content elicited in the absence of Ppb1 activity. Contrariwise, the enhancement of CIP activity achieved by the overexpression of a constitutively active version of Pek1 MAPKK further increased the cytoplasmic calcium levels in a Yam8/Cch1-dependent fashion [135]. As a whole, these findings suggest that both the calcineurin and the CIP pathways may antagonistically regulate calcium uptake in fission yeast through the fine-tuning of the dephosphorylation/phosphorylation status of the Yam8–Chh1 channel (Figure 1D) [135]. Hence, the *vic* phenotype, which was initially associated with altered chloride homeostasis, is actually due to the suppression by CIP inhibition of the lethal effect resulting from the persistent uptake of calcium via Yam8–Chh1 during salt stress and in the absence of calcineurin activity. This model supports that calcineurin activity is dominant over CIP signaling during unperturbed growth, and, therefore, the Yam8–Cch1 channel remains basically inactive. Conversely, in response to salt stress, activated Pmk1 may phosphorylate and promote the Cch1–Yam8 opening and Ca^+2^ influx. In turn, the increase in intra-cytoplasmic calcium levels triggers calcineurin activation, resulting in the dephosphorylation and closure of the channel (Figure 1D) [135]. Formal confirmation of this regulatory mechanism will require the demonstration of a direct physical association of Pmk1 and Ppb1 with Yam8–Cch1 and the identification of the putative conserved residues that become phosphorylated or dephosphorylated by the kinase and phosphatase, respectively, to modulate the activity of the channel depending on the environmental context.

### 3.3. Cell-Wall Integrity

The fungal CW, largely composed of glucans, mannans, and chitin, is an essential barrier that protects and maintains cellular integrity during polarized vegetative growth and other morphogenetic processes such as mating, sporulation, or pseudohyphal growth [136]. The CW is a highly dynamic structure that adjusts its composition and structure in response to environmental changes (pH, temperature shifts, carbon source, etc.), or during drug exposure and immune responses [136,137]. The CW must be assembled to become both malleable and mechanically robust, in order to withstand the elevated cytoplasmic turgor pressure and adequately perform its biological functions. These adaptive and compensatory mechanisms involve different strategies depending on the fungi, but their ultimate goal is to repair the damaged/altered CW and guarantee cell survival. Although the CIP lies at the core of such responses, both in budding and fission yeasts, the molecular processes involved are known with greater detail in the former species [31,33].

Most fungal CWs are composed of an innermost and relatively conserved layer that functionally represents the load-bearing component of the wall (glucans and chitin), while the outer layers are more variable and specifically tailored to suit the needs of each particular species [136]. In *S. pombe*, most of the CW is composed of branched β-1,3-glucan synthesized by the membrane-bound β-1,3-glucan synthase (β-GS) complex. During division, a three-layered septum is formed with the central area being made of linear β-1,3-glucan surrounded by two outer secondary septa [33]. The CW behaves differently at the tips during polarized growth as compared to the cell sides and exhibits a fluctuating behavior with thickening phases followed by thinning phases. On the contrary, the cell sides remain stable [35]. Therefore, the tips are twice as soft and thin with respect to the remainder of the cell. Importantly, CW thickness is positively regulated by synthesis and negatively regulated by growth [35].

In *S. pombe*, the essential GTPase Rho1 is the β-GS regulatory subunit responsible for the activation of GS catalytic subunits Bgs1 to Bgs4 [41,53], likely through conformational changes dependent on their direct physical binding, as described in *S. cerevisiae* [138]. In addition, Rho1 activates β-1,3-glucan biosynthesis indirectly through the CIP components and PKC orthologs, Pck1 and Pck2 [55,56]. On the other hand, Rho2 activates Mok1 [45], which is the essential enzyme responsible for CW α-1,3-glucan synthesis, via Pck2 [33]. How Rho1 and Rho2 activities are precisely controlled in response to internal or external cues to dynamically modulate CW structure remains an open question, but it likely involves the regulation of the activity of their specific GEFs and/or GAPs [38]. As previously noted, the fission yeast sensors Wsc1 and Mtl2 physically couple the CW with the plasma membrane to activate Rho1 and CW biogenesis through its interaction with Rgf1 GEF and, to a lesser extent, Rgf2 [32,35]. However, the data obtained so far suggest that Wsc1 and Mlt2 may perform this function independently of the CIP [32]. These findings support that mechanosensing through these sensors might not be the major mechanism responsible for Rho- and PKC-dependent CIP activation, which mainly relies on the Rho2–Pck2 branch. Besides, Wsc1 clustering at the membrane does not depend on any members of CIP, including Pmk1 MAPK [37]. Even so, Pmk1 has been involved in the homeostatic maintenance of CW thickness during polarized cell growth by adjusting synthesis-to-surface growth in order to avoid excessive thinning [35]. However, the molecular mechanisms involved in this control are far from being understood. As mentioned earlier, the only transcriptional CW-related target for the Pmk1–Atf1 branch is the *ecm33^+^* gene, encoding a GPI-anchored cell-surface protein [97]. This makes it rather unlikely that the CIP may play a direct and significant role during the control of CW homeostasis at a transcriptional level. Nevertheless, Pmk1 might affect CW integrity through alternative pathways, such as regulating calcium influx in response to CW damage through the Yam8–Cch1 channel and involving the calmodulin/Prz1-dependent transcriptional response or by exerting regulatory feedbacks on the Rho-PKC module to elicit CW synthesis and/or CIP signaling. In budding yeast, it has been suggested that the MAPK Mpk1/Slt2 activates a negative feedback loop that downregulates CIP signaling by detaching Rho1 from its GEF Rom2 [139]. A recent study has shown that constitutive activation of the CIP in cells grown in heavy water (D_2_O), causes gross morphological changes, thickened CWs, and aberrant septa that limit cell growth [140]. Remarkably, blocking CIP activity partially alleviates those phenotypes, while its activation increases cellular sensitivity to D_2_O [140]. Moreover, it has been recently described that moderate cell-wall stress extends chronological lifespan in fission yeast in a Pmk1-dependent manner [141]. Theis evidence supports the fact that the activity of the CIP must be precisely regulated in time and space to preserve CW integrity and cellular viability.

Another remarkable difference between budding and fission yeasts with regard to the functional role of the CIP in CW biogenesis relates to the control of the actin cytoskeleton. In *S. cerevisiae*, Rho1 is important for proper actin cytoskeleton organization through the control of the CIP MAPK Mpk1/Slt2 [31]. However, such a functional link has not been established so far in fission yeast, and the regulatory mechanisms underlying actin cytoskeleton reorganization and integrity in response to environmental stimuli are mostly unknown. Future studies in *S. pombe* will help to elucidate the details of the possible relevance of Rho GTPases and the CIP during actin cytoskeleton organization.

As discussed earlier, the CIP plays a homeostatic role during the control of polarized cell growth [35]. In fission yeast, the Cdc42 GTPase module is a major regulator of polarized growth, which involves intertwined connections between surface material synthesis by exocytosis and endocytosis, the actin cortex, and the CW [38]. Considering that polarized growth may be counteracted by CW stiffness, the existence of functional crosstalk between the Cdc42 polarity network and the CIP should be expected. In this regard, it was suggested in early studies that Cdc42 might signal through the CIP pathway via the redundant PAK kinase Pak2, since the expression of a dominant-activated *cdc42G12V* allele prompted a strong growth defect that was rescued by both *pak2Δ* and *mkh1Δ* deletions [142]. Unexpectedly, neither *pak2^+^* deletion nor *cdc42G12V* overexpression had a noticeable effect on Pmk1 activity during growth and stress [18]. Cdc42 could regulate a minimal subset of CIP components localized at the growing poles, which might hamper their identification in the studies performed so far. Another possibility is that the CIP Rho GTPases and/or PKCs might be targeted by Cdc42 to modulate CW homeostasis independently of their role during CIP signaling [38]. In agreement with this hypothesis, a functional relationship between the polarity machinery and the Rho1–Pkc1 function has been described in *S. cerevisiae* [143].

Altogether, these findings support that the CIP plays a determinant role in maintaining and modulating cell-wall integrity in fission yeast during vegetative growth and in response to different stimuli, although further studies will be required to shed light onto the detailed molecular mechanisms and processes responsible for such control.

### 3.4. Cytokinesis

Cytokinesis, the final stage of cell division, involves the assembly and constriction of a contractile actomyosin ring (CAR) that physically divides the mother cell into two daughter cells. Contrary to most animal and fungal cells, including fission yeast *S. japonicus*, which assemble the CAR when chromosomes are fully segregated, CAR assembly in *S. pombe* is a rather counterintuitive biological phenomenon that starts early in anaphase before chromosomal segregation takes place [144]. Fission yeast cytokinesis can be divided into four distinct stages: (i) node condensation and CAR assembly; (ii) CAR maturation; (iii) CAR constriction and septum synthesis; and iv) septum digestion and cell separation. First, pre-nodes containing the anillin-like protein Mid1 and cell-cycle kinases evolve into cytokinetic nodes through the addition of myosin II Myo2, IQGAP Rng2, F-BAR protein Cdc15, and the formin Cdc12, which together with For3, another formin, stimulate the nucleation of actin filaments [145]. The subsequent and dynamic interactions between myosin II and the actin filaments promote node condensation for assembly of the CAR. The CAR maturation stage involves the recruitment to the CAR of Cdc15 and several other proteins, including Rga7 (Rho2 GAP) and Rgf3 (Rho1 GEF) [145]. Later, the CAR constricts by the action of the motor type II myosin Myp2 and cofilins, which cut actin fragments stochastically to shorten the ring [145]. CAR constriction and the synthesis of a new CW via the formation of a three-layered septum is triggered by the Septation Initiation Network (SIN) cascade, which is related to the Hippo and MEN signaling pathways in mammals and *S. cerevisiae*, respectively [111]. Finally, cell separation liberates the two daughter cells upon dissolution of the primary septum through the specific action of the glucanases Eng1 and Agn1 localized at the division site [146].

In *S. cerevisiae* the CIP regulates mechanistically distinct aspects of cytokinesis both at the transcriptional and post-transcriptional levels. During late mitosis, Polo-activated Rho1 mediates the assembly of the actomyosin ring through the formin Bni1, a fission yeast Cdc12 ortholog [147], which leads to deposition of the primary septum by the chitin synthase Chs2 [148]. Right after mitosis, Rho1 also activates the chitin synthase Chs3 and the β-glucan synthases Fks1/2 that participate in secondary septum biogenesis via Pkc1 [149]. Activation of the CIP and Mpk1/Slt2 MAPK by Rho1 is also biologically relevant for the transcriptional induction of several cytokinetic genes, such as Fks2 (glucan synthase), Gfa1 (required for the synthesis of a metabolic precursor of chitin), and Chr1/2 (transglycosidases) [150,151]. Contrariwise, the CIP-dependent transcriptional response seems rather irrelevant during cytokinesis control in fission yeast. However, similar to budding yeast, several studies have highlighted the key role of fission yeast Rho1, and Rho2, in the activation of glucan synthases during primary and secondary septum deposition (reviewed in [38]).

The assembly of a septin ring, which is one of the first cytokinetic events in budding yeast, is modulated by the CIP under specific circumstances [152]. In this regard, it has been shown that the ER Stress Surveillance (ERSU) pathway, a mechanism that safeguards the inheritance of a functional endoplasmic reticulum (ER) by the daughter cell, is regulated by Mpk1/Slt2 signaling [152]. Upon ER stress, both ER inheritance and cytokinesis are inhibited due to the relocalization of the septin complex away from the bud neck [153]. However, in the absence of Mpk1/Slt2, cells do not block ER inheritance, and the septin ring remains positioned at the bud neck [152]. The putative functional connection between ER stress, the CIP, and septum assembly has not been explored so far in fission yeast. Nevertheless, several studies point to a functional role of Pmk1 during CAR assembly and maturation that influences septal dynamics in response to several stressors, although the precise molecular details remain obscure [110,154]. As described earlier, the Pmk1-dependent phosphorylation of the RNA-binding protein Nrd1 negatively regulates its ability to bind and stabilize *cdc4^+^* mRNA, thus allowing for the accurate regulation of myosin II function during CAR assembly and constriction (Figure 1C) [110]. In addition, recent data suggest that the CIP may participate in a checkpoint that imposes a delay during the maturation phase of CAR assembly upon CW-integrity stress [154]. This inhibition is exerted through Rgf1–Rho1 activation of the CIP components Pck2 and Pmk1 and ensures that cytokinesis is completed after cells have adapted to the new growth conditions [154].

*S. pombe* mutants lacking CIP components display distinct phenotypic penetrance during cytokinesis. The upstream CIP GTPase members, Rho1 and Rho2, and PKC orthologs, Pck1 and Pck2, play an essential role in this process, as either Rho1 downregulation or simultaneous deletion of Pck1 and Pck2 produce strong cytokinetic defects that result in cell death [6]. Rho1 (and Rho2) plays a prominent role during septum synthesis, whereas Rho3 and Rho4 participate in the dissolution step [38]. It has been proposed that the SIN pathway may target the Rho1 GEF Rgf3 to promote septation [155], although this has not been formally proven. Rho1 activation at the CAR by Rgf3 is essential for proper ring maturation and constriction [59,156]. In addition to this, low levels of Rgf3 at the division site cause septum defects and cell lysis due to poor β-GS activity [156]. On the other hand, CIP mutants lacking the MAPK module components Mkh1, Pek1, and Pmk1 show mild separation defects, probably due to late defects in CW-remodeling that become exacerbated under stress. Furthermore, Pmk1 hyperactivation also leads to cell separation defects [6,74]. These phenotypes might be partially related, as previously mentioned, to the activity of the Pmk1–Nrd1 regulatory axis [110]. Alternatively, Pmk1 might be necessary for the activation at the CAR of calcineurin, which participates in the dephosphorylation of the F-BAR protein Cdc15 during cytokinesis [157].

Activation of the CIP pathway in budding yeast induces the phosphorylation of eisosome core components such as Pil1 and Lsp1 [158], whereas Pmk1 activation in fission yeast is inhibited by eisosomes [48]. These observations raise the possibility that Pmk1-dependent regulation of eisosome dynamics might alter plasma membrane compartmentalization, thus affecting the endocytosis of proteins involved in septum assembly. In this regard, the CIP components Rgf1, Pck1, Pck2, and Pmk1 cooperate to localize the polarity landmarks Tea1, Tea4, and Pom1 at the cell cortex; it has been suggested that the CIP likely regulates membrane–lipid homeostasis [159]. The possibility that CIP signaling impacts protein turnover at the membrane, suggests that its role during polarized growth and cytokinesis will surely be investigated further in the future. Finally, *S. cerevisiae* mutants with defects in Chs2 function, or in the actomyosin and septin rings, are able to assemble abnormal and engrossed septa (known as “remedial septa”), to prevent lysis during cytokinesis. Importantly, these situations trigger a strong CIP activation that results in the up-regulated synthesis of Gfa1, Fkh1/2, and Chr1/2 [150]. Remedial septa have also been described in *S. pombe* mutants that are defective in proper septum assembly [160], but the involvement of the CIP pathway during their formation has not been depicted.

The evidence obtained so far suggests that the CIP has a significant impact on fission yeast cytokinesis, where the cell wall provides the strength to generate the invaginating furrow. However, this contribution might have been overlooked, as the CAR and septal dynamics have been studied during unperturbed growth in most studies.

## 4. Functional Crosstalks of the CIP

### 4.1. Interplay with Other Fission Yeast MAPK-Signaling Cascades

*S. pombe* only has two conserved MAPK cascades besides the CIP, the mating pheromone-responsive pathway, and the stress-activated MAP kinase (SAPK) pathway [14,15,17,90,161,162,163]. While MAPK cascades are evolutionarily organized in a strict and modular fashion to achieve signal specificity in response to external cues, some core components (i.e., kinases, scaffolds) are sometimes shared by different MAPK cascades, resulting in an enhanced signaling plasticity and a more precise coordination of biological responses. This situation does not seem to occur in fission yeast, as the main components of its three MAPK modules and their corresponding upstream activators are pathway specific. However, some downstream elements of the CIP are shared by the stress- and pheromone-signaling cascades [18,91,164]. These include the Atf1 transcription factor and several MAPK phosphatases (Pyp1, Pyp2, Ptc1, Ptc2, and Ptc3), together with RNA-binding proteins (Rnc1 and Nrd1).

Early observations pointed out the existence of a strong functional crosstalk between the CIP and the SAPK pathways during the stress response in fission yeast. Sty1 MAPK, which is the ortholog of budding yeast Hog1 and mammalian p38, plays a crucial role during fission yeast survival in response to multiple forms of stress [88,90,91,162,163,165,166,167,168,169]. Similar to the SAPK pathway, the CIP becomes activated to multiple stressful conditions [18]. However, while some stimuli specifically activate Pmk1 (CW stress) or Sty1 (nitrogen starvation), many others, such as oxidative stress, hyperosmotic stress, thermal stress, glucose depletion, and hypergravity, prompt the simultaneous activation of both pathways [6].

Various pro-oxidants and oxidizing agents such as hydrogen peroxide, diamide, diethyl maleate (DEM), Paraquat, and TBH, induce activation in both Sty1 and Pmk1 [18,167,170]. However, whereas Sty1- or Atf1-deleted mutants are strongly growth-sensitive for growth in the presence of these compounds [167], the sensitivity of Pmk1-less mutants is minimal [18]. Sty1 is activated in response to both low and high concentrations of hydrogen peroxide [167], but Pmk1 activation only occurs at high concentrations of the oxidant (>5 mM) [18]. These observations support the fact that the biological significance of the CIP pathway during the defensive response against oxidative stress is rather low compared with the SAPK pathway. From a general perspective, the differences in the respective activation patterns of both MAPKs and the fact that the overall stress-sensitivity of mutants devoid of Sty1 is higher than in *pmk1Δ* cells have led to the suggestion that the CIP pathway may reinforce the SAPK pathway to control cell survival and adaptation to sub-lethal stressful conditions [6,18].

Deletion of Pmk1 does not affect Sty1 activation in response to hyperosmotic stress [18]. However, a lack of the SAPK MAPK module components, including Sty1, increased both the basal and the stress-induced levels of Pmk1 phosphorylation [20]. Moreover, accurate deactivation of Pmk1 following osmostress required a functional Sty1–Atf1 transcriptional branch [18], suggesting that one or more MAPK phosphatases induced through the SAPK pathway might be responsible for Pmk1 deactivation during growth and stress. Negative regulation of Sty1 activity is carried out by tyrosine phosphatases Pyp1 and Pyp2 and serine/threonine phosphatases Ptc1 and Ptc3 [171]. Pyp1 is the main phosphatase deactivating Sty1 during unperturbed growth [90,172], and both Pyp1 and Pyp2 dephosphorylate Sty1 activated under osmotic or oxidative stress [162,163]. During heat shock, Pyp1 binding to Sty1 is abolished, resulting in a quick and strong Sty1 activation that is subsequently attenuated by Ptc1 and Ptc3 [173]. The basal expression of *pyp1^+^* and the stress-induced expression of *pyp1^+^*, *pyp2^+^*, and *ptc1^+^* are regulated by Sty1 and Atf1 through negative feedback loops [90,91,92,174]. Significantly, these three phosphatases have been shown to associate with and dephosphorylate Pmk1 in vivo during growth and stress (Figure 1E) [20]. It has been reported that Ptc3 is also involved in Pmk1 down-regulation [96]. Pyp1 and Ptc1 control Pmk1 basal activity during unperturbed vegetative growth and Pmk1 hyperactivation during cellular adaptation to osmostress is reduced by Pyp1, Pyp2, and Ptc1 [20]. In support of this model, cells devoid of the Receptor of Activated C Kinase (RACK1) ortholog Cpc2, which binds to the ribosomal 40S subunit and is essential for efficient up-regulation of Pyp1 and Pyp2 translation, show enhanced Pmk1 activation as a result of a decrease in the protein levels of both phosphatases [118].

Finally, the dual-specificity phosphatase Pmp1/Dsp1, which is the main negative regulator of Pmk1 activity during vegetative growth [25], also downregulates the MAPK of the pheromone response pathway Spk1 (Figure 1E) [164]. Pmp1 is closely related to the budding yeast dual-specificity phosphatase Msg5, which was isolated as a suppressor of the pheromone-signaling pathway that dephosphorylates Fus3 MAPK [175]. Msg5 also negatively regulates Mpk1/Sst2 and Kss1, which are the respective MAP kinases of the cell-integrity and filamentation/invasion pathways [176,177,178]. Hence, the substrate promiscuity of dual-specificity MAPK phosphatases is likely a common theme in the group of yeast MAPK cascades.

### 4.2. CIP and TOR Signaling Crosstalk

The Target of Rapamycin (TOR) is a conserved Ser/Thr kinase that regulates cell growth and metabolism in response to environmental cues [179]. TOR is found as two evolutionary conserved complexes, TORC1 and TORC2 [180,181,182]. In fission yeast, TORC1 is essential for vegetative growth and regulates the switch between cell proliferation and differentiation by sensing nutrient availability [183,184,185]. In contrast to TORC1, TORC2 is dispensable for proliferation under optimal growth conditions but is required for cellular adaptation during starvation and in response to stress [11,186]. The AGC-family kinases Psk1 and Gad8 are two direct targets for TORC1 and TORC2, respectively [184,187,188]. Notably, similar to Pck2, which is the main upstream kinase activator of the CIP MAPK module [78], the activity of both Gad8 and Psk1 is dependent upon AL phosphorylation mediated by the PDK1 ortholog Ksg1 [184,187].

For budding and fission yeasts, TOR and MAPK pathways play essential cellular functions in response to environmental changes in budding and fission yeasts; therefore, it is not surprising that both pathways are functionally interconnected to a large extent [11,72,189,190,191,192,193]. In *S. pombe*, a highly intricate cross-regulatory relationship between the TORC2 complex and the CIP secures cell adaptation and survival in response to environmental cues (Figure 1E) [72]. Increased de novo synthesis of Pck2 is essential to trigger Pmk1 activation in response to CW damage or upon glucose limitation. TORC2 positively controls this process through its main activator, the Rab GTPase Ryh1 [72]. GTP-Ryh1 associates with TORC2 to induce the phosphorylation and activation of Gad8 in glucose-rich media, and its activity is strongly reduced under glucose deprivation [194]. Moreover, Ryh1 modulates the CIP via TORC2-dependent and -independent mechanisms. In the TORC2-dependent mechanism Gad8, with the participation of TORC1 target Psk1, there is an increase in Pck2 protein levels that contributes to Pmk1 activation during CW stress and glucose limitation (Figure 1E) [72]. Interestingly, the inhibition of TORC1 in *S. pombe* by nutrient deprivation induces the phosphorylation of the translation initiation factor eIF2α [185], which negatively regulates translation in response to different environmental challenges [195,196,197]. Psk1 might reinforce Pck2 synthesis during stress by maintaining low eIF2α phosphorylation levels. Nevertheless, the upregulation of Pck2 synthesis by Gad8–Psk1 kinases does not operate through the phosphorylation of Rps6, an evolutionary conserved ribosomal protein, which is phosphorylated in vivo by both Psk1 and Gad8 in response to nitrogen and glucose sources [72,198,199]. Further, Ryh1 may prompt Pmk1 activation, regardless of its role as an upstream regulator of TORC2 signaling, by promoting proper plasma membrane tethering and/or processing of several key activators of the CIP, including Ksg1, Rho1, Rho2, Rgf1, and Pck2 (Figure 1A,E). This alternative mechanism is particularly relevant during MAPK activation in response to osmotic stress. The novel role of Ryh1 as elicitor of MAPK signaling does not seem to be conserved in budding yeast, since the deletion of Ypt6 (Ryh1 ortholog) did not avoid stress-activation of the respective CWIs and p38 MAPKs Slt2 or Hog1 [72]. Moreover, the phosphatidylinositol (PI) 4-phosphate 5-kinase orthologs Its3 and PI(4,5)P2, which promote Ryh1-TORC2 signaling, also act downstream Ryh1 to regulate trafficking and localization of several CIP activators of the CIP cited above (Figure 1E) [72]. The dual functional role of PI kinases might be evolutionarily conserved, since the budding yeast PI kinases also regulate PDK orthologs Pkh1/2 and TORC2 at the plasma membrane [200].

Glucose starvation results in the activation of Pmk1 through TORC2-dependent and -independent mechanisms. TORC2 activity, as measured using the in vivo phosphorylation status of Gad8 at S-546 as a readout, decreases quickly but recovers to half of the initial levels after 30 min of incubation and remains unchanged for longer times [11,72,194]. Importantly, CIP signaling inhibits TORC2-dependent Gad8 phosphorylation and activation during glucose starvation and CW stress. Accordingly, Gad8 phosphorylation at S-546 was quickly reduced in wild-type and *pmk1Δ* cells upon both stresses and increased significantly in the *pmk1Δ* mutant at longer times [11,72]. It has been reported that Pck2 phosphorylates Gad8 in vivo to downregulate TORC2–Gad8 signaling [201]. However, the Pmk1 negative regulation of TORC2 activity does not rely on Pck2 and/or Gad8. The reduction of Ryh1 GTPase activity and Gad8-S546 phosphorylation levels displayed by fission yeast cells during prolonged incubation without glucose became significantly attenuated in a *pmk1Δ* background. This led to the hypothesis that Pmk1 may negatively modulate TORC2–Gad8 signaling in response to glucose deprivation and likely CW stress, by downregulating the Ryh1 GTPase activation cycle (Figure 1E) [72].

Hyperosmotic stress transiently inhibits TORC2 activity independent of the function of the SAPK MAPK Sty1 [191,202]. However, Sty1 promotes TORC2 reactivation after osmostress through the transcription factor Atf1 and the glycerol-3-phosphate dehydrogenase Gpd1, whose expression was induced by the SAPK pathway [191]. Although CIP signaling was involved in the negative regulation of the TORC2–Gad8 pathway under osmotic saline stress conditions [11,72], the evidence obtained so far suggests that Pmk1 is not required for the osmo-inhibition of TORC2 signaling [191].

### 4.3. Interaction between the CIP and Camp/PKA Pathways

Evidence points to the existence of a functional crosstalk between the CIP and cAMP/PKA pathways in different fungal models. For example, alterations of the β-1,3-glucan network in budding yeast induce both CIP activation via Wsc1 and the parallel inhibition of cAMP/PKA signaling [203]. Although cAMP levels were reduced during CW damage induced using caspofungin independent of Slt2 activation, both responses are required to counteract the detrimental effects of the drug [203]. Further, deletion of *Cryptococcus neoformans* genes encoding the core kinases in the CIP pathway (namely, Pkc1, Bck1, Mkk2, and Mpk1) results in severe cell wall defects, including a loss of surface capsule and decreased virulence in a mouse model [204]. Interestingly, in these mutants, capsule formation is impaired due to reduced cAMP levels, and this defect is suppressed by the addition of exogenous cAMP [205]. These data suggest the existence of an intricate functional network between the CIP and the cAMP/PKA pathways, which is relevant for the appropriate regulation of fungal cell wall homeostasis.

The fission yeast CIP has also been shown to functionally interact with cAMP/PKA signaling [11,83]. In this organism, the cAMP/PKA pathway is involved in the control of relevant cellular events, including glucose sensing, sexual differentiation, spore germination, and osmotic stress response [206,207]. The pathway starts with the detection of extracellular glucose by the G-protein coupled receptor Git3. The signal is then transmitted to a membrane-bound adenylate cyclase Cyr1/Git2 through a heterotrimeric G protein composed of the Gpa2 Gα, the Git5 Gβ, and the Git11 Gγ subunits. Cyr1 triggers an increase in cAMP levels, which prompts the activation of the Pka1 catalytic subunit through its dissociation from the regulatory subunit Cgs1 [208]. Pka1 phosphorylates and negatively regulates the activity of Rst2, a transcription factor responsible for the induced expression of genes, such as *fbp1*^+^, encoding the gluconeogenic enzyme fructose-1,6-bisphosphatase, whose activity is critical for adaptive growth to non-fermentable carbon sources (i.e., in the absence of glucose) [206]. Pmk1 becomes activated upon glucose deprivation, but the magnitude of this response is reduced in the absence of a cAMP/PKA function, as observed in *git3Δ*, *gpa2Δ*, *cyr1Δ*, and *pka1Δ* mutants. Contrariwise, Pmk1 activation in response to glucose starvation remained unchanged in cells lacking the Pka1-dependent transcriptional regulator Rst2 [83]. These findings suggest that an operative cAMP/PKA pathway is necessary for the full activation of the CIP under glucose limitation, but this control is exerted in a Rst2-independent way. However, the detailed responsible molecular mechanisms involved in this crosstalk wait to be elucidated.

In summary, although the SAPK has arguably been considered the most prominent and physiologically relevant MAPK pathway in fission yeast, it is becoming increasingly evident that the functional interactions of the CIP with additional MAPKs and TOR and PKA signaling cascades are also essential for this organism to integrate multiple adaptive responses to the changing environment.

## 5. Concluding Remarks and Perspectives

CIP signaling is involved in the regulation of essential cellular processes in fission yeast, such as the RBPs-mediated control of mRNA-stability, ionic homeostasis, CW integrity, and cytokinesis. Studies completed during the last 30 years have depicted the complexity and pleiotropism of environmentally controlled CIP signaling during the fission yeast life cycle, which is likely due to the existence of a wide number of effector substrates. However, very few of these putative targets have been identified to date. Therefore, their discovery will help to clarify and expand the repertoire of cellular processes regulated by this MAPK cascade. Another area of future investigation that surely deserves further deepening relates to the elucidation of the precise molecular mechanisms responsible for the functional interaction of the CIP with other environmentally regulated signaling pathways. Ultimately, comprehension of how CIP activity modulates fission yeast cellular responses might be compared to conserved pathways present in other fungal models and also in higher eukaryotes.

## Figures and Tables

**Table 1:** Main functional roles of fission yeast CIP components.

	Protein	Function	*S. cerevisiae* Ortholog
Cell-surface sensors	Wsc1	- Plasma membrane-associated serine-rich cell wall mechanosensor located at active growth sites and the division septum - Detects perturbations at the CW and plasma membrane and transmits signals through Rgf1 (Rho1 GEF)	WSC1
	Mtl2	- Plasma membrane-associated serine-rich cell wall mechanosensor located at cellular periphery - Detects perturbations at the CW and plasma membrane and transmits signals through Rgf1	MLT1
Regulators of GTPases	Rgf1	- Guanine nucleotide exchange factor (GEF) for Rho1 GTPase - Activates the CIP via Rho1 and Pck2	ROM1
	Rga2	- GTPase activating protein (GAP) for Rho1 and Rho2. - Negatively regulates the Rho2–Pck2 interaction with the CIP	BEM3
	Rga4	Rho2 GAP that negatively regulates the activity of the CIP	RGA2
	Rga6	Rho2 GAP	
	Rga7	Rho2 GAP that negatively regulates the activity of the CIP	RGD1
Rho GTPases	Rho1	- Regulation of cell wall biosynthesis by β-glucan synthases Bgs1-4 - Stabilizes Pck1 and Pck2 - Regulates the CIP in response to cell wall damage, independently of Wsc1 and Mlt2 - Cytokinesis checkpoint to cell wall damage through Pmk1	RHO1
	Rho2	- Regulation of cell wall biosynthesis by α-glucan synthase Mok1/Ags1 - Major CIP regulator together with Pck2	RHO2
	Rho4	- Minor role in CIP signaling	RHO4
	Rho5	- Functional paralogue of Rho1 - Minor role in CIP signaling	RHO1
Phosphoinositide metabolism	Ksg1	Serine/threonine protein kinase PDK-1ortholog involved in CIP signaling through the activation of Pck1 and Pck2	PKH1/PKH2
PKCs and the CIP MAPK cascade	Pck1	Rho1 target involved in CIP signaling in response to CW damage	PKC1
	Pck2	-Rho1 and Rho2 target - Main upstream activator of the CIP MAPK module during growth and stress	PKC2
	Mkh1	MAPK kinase kinase	BCK1
	Pek1	MAPK kinase	MKK1/MKK2
	Pmk1	MAPK	MPK1/SLT2
Negative regulators	Skb5	-Shk1 kinase binding protein - Inhibits Pmk1 by downregulating Mkh1 localization to cell tips	NBP2
	Pmp1	- Dual-specificity MAP kinase phosphatase - Binds and dephosphorylates Pmk1 during vegetative growth	SDP1/MSG5
	Pyp1	Tyrosine phosphatase: - Transcriptionally regulated by the SAPK pathway (Sty1-Atf1) - Binds and dephosphorylates both Sty1 and Pmk1 during vegetative growth	PTP3, PTP2
	Pyp2	Tyrosine phosphatase: - Transcriptionally regulated by the SAPK pathway (Sty1-Atf1). - Binds and dephosphorylates both Sty1 and Pmk1 during stress	PTP3, PTP2
	Ptc1	Serine/threonine phosphatase: - Transcriptionally regulated by the SAPK pathway (Sty1-Atf1). - Binds and dephosphorylates both Sty1 and Pmk1 during vegetative growth	PTC1
	Ptc3	Serine/threonine phosphatase: - Transcriptionally regulated by the SAPK pathway (Sty1-Atf1). - Binds and dephosphorylates both Sty1 and Pmk1 during stress	PTC3, PTC2
Downstream targets	Atf1	- Atf-CREB family bZIP domain transcription factor - Downstream effector of Sty1 MAPK(SAPK pathway) in response to environmental cues and Pmk1 in response to CW damage	---
	Nrd1	RNA-binding protein (RBP): - Phosphorylated by Pmk1 during growth and stress - Negatively modules myosin II essential light chain mRNA stability - Regulation of actomyosin ring integrity during cytokinesis	MRN1
	Rnc1	KH domain RBP: - Phosphorylated by Pmk1 during growth and stress - Phosphorylated by Sty1 during growth and stress - Stabilizes mRNA encoding Pmk1 dual specificity phosphatase Pmp1 - Negative regulation of Pmk1 activity	PBP2
	Clp1	Cdc14-related serine/threonine protein phosphatase: -Phosphorylated by Pmk1 - Pmk1-dependent phosphorylation promotes its nucleoplasmic accumulation during genotoxic stress	CDC14
	Cch1- Yam8	Plasma-membrane channel complex: - Putative substrate for Pmk1 phosphorylation - Putative substrate for dephosphorylation by Calcineurin (Ppb1) - Positively regulates import of calcium ions in response to salt stress	CCH1-MID1

## References

[1] Errede B., Levin D.E. (1993). A conserved kinase cascade for MAP kinase activation in yeast. Curr. Opin. Cell Biol..

[2] Marshall C.J. (1995). Specificity of receptor tyrosine kinase signaling: Transient versus sustained extracellular signal-regulated kinase activation. Cell.

[3] Bluthgen N., Legewie S. (2008). Systems analysis of MAPK signal transduction. Essays Biochem..

[4] Waskiewicz A.J., Cooper J.A. (1995). Mitogen and stress response pathways: MAP kinase cascades and phosphatase regulation in mammals and yeast. Curr. Opin. Cell Biol..

[5] Marshall C.J. (1994). MAP kinase kinase kinase, MAP kinase kinase and MAP kinase. Curr. Opin. Genet. Dev..

[6] Pérez P., Cansado J. (2010). Cell integrity signaling and response to stress in fission yeast. Curr. Protein Pept. Sci..

[7] Kyriakis J.M., Avruch J. (2012). Mammalian MAPK signal transduction pathways activated by stress and inflammation: A 10-year update. Physiol. Rev..

[8] Hill C.S., Treisman R. (1995). Transcriptional Regulation by Extracellular signals: Mechanisms and Specificity. Cell.

[9] Krishna M., Narang H. (2008). The complexity of mitogen-activated protein kinases (MAPKs) made simple. Cell. Mol. Life Sci..

[10] Lavoie H., Gagnon J., Therrien M. (2020). ERK signalling: A master regulator of cell behaviour, life and fate. Nat. Rev. Mol. Cell Biol..

[11] Cohen A., Kupiec M., Weisman R. (2014). Glucose activates TORC2-Gad8 protein via positive regulation of the cAMP/cAMP-dependent protein kinase A (PKA) pathway and negative regulation of the Pmk1 protein-mitogen-activated protein kinase pathway. J. Biol. Chem..

[12] Schaeffer H.J., Weber M.J. (1999). Mitogen-activated protein kinases: Specific messages from ubiquitous messengers. Mol. Cell. Biol..

[13] Shiozaki K., Russell P. (1995). Cell-cycle control linked to extracellular environment by MAP kinase pathway in fission yeast. Nature.

[14] Toda T., Shimanuki M., Yanagida M. (1991). Fission yeast genes that confer resistance to staurosporine encode an AP-1-like transcription factor and a protein kinase related to the mammalian ERK1/MAP2 and budding yeast FUS3 and KSS1 kinases. Genes Dev..

[15] Zaitsevskaya-Carter T., Cooper J.A. (1997). Spm1, a stress-activated MAP kinase that regulates morphogenesis in *S.pombe*. Embo J..

[16] Sugawara T., Sato M., Takagi T., Kamasaki T., Ohno N., Osumi M. (2003). In situ localization of cell wall alpha-1,3-glucan in the fission yeast *Schizosaccharomyces pombe*. J. Electron. Microsc..

[17] Toda T., Dhut S., Superti-Furga G., Gotoh Y., Nishida E., Sugiura R., Kuno T. (1996). The fission yeast pmk1+ gene encodes a novel mitogen-activated protein kinase homolog which regulates cell integrity and functions coordinately with the protein kinase C pathway. Mol. Cell. Biol..

[18] Madrid M., Soto T., Khong H.K., Franco A., Vicente J., Pérez P., Gacto M., Cansado J. (2006). Stress-induced response, localization, and regulation of the Pmk1 cell integrity pathway in Schizosaccharomyces pombe. J. Biol. Chem..

[19] Loewith R., Hubberstey A., Young D. (2000). Skh1, the MEK component of the mkh1 signaling pathway in Schizosaccharomyces pombe. J. Cell Sci..

[20] Madrid M., Núñez A., Soto T., Vicente-Soler J., Gacto M., Cansado J. (2007). Stress-activated protein kinase-mediated down-regulation of the cell integrity pathway mitogen-activated protein kinase Pmk1p by protein phosphatases. Mol. Biol. Cell.

[21] Garcia P., Tajadura V., Sanchez Y. (2009). The Rho1p exchange factor Rgf1p signals upstream from the Pmk1 mitogen-activated protein kinase pathway in fission yeast. Mol. Biol. Cell.

[22] Sugiura R., Toda T., Dhut S., Shuntoh H., Kuno T. (1999). The MAPK kinase Pek1 acts as a phosphorylation-dependent molecular switch. Nature.

[23] Viana R.A., Pinar M., Soto T., Coll P.M., Cansado J., Pérez P. (2013). Negative functional interaction between cell integrity MAPK pathway and Rho1 GTPase in fission yeast. Genetics.

[24] Sengar A.S., Markley N.A., Marini N.J., Young D. (1997). Mkh1, a MEK kinase required for cell wall integrity and proper response to osmotic and temperature stress in Schizosaccharomyces pombe. Mol. Cell. Biol..

[25] Sugiura R., Toda T., Shuntoh H., Yanagida M., Kuno T. (1998). pmp1+, a suppressor of calcineurin deficiency, encodes a novel MAP kinase phosphatase in fission yeast. EMBO J..

[26] Ma Y., Kuno T., Kita A., Asayama Y., Sugiura R. (2006). Rho2 is a target of the farnesyltransferase Cpp1 and acts upstream of Pmk1 mitogen-activated protein kinase signaling in fission yeast. Mol. Biol. Cell.

[27] Qi M., Elion E.A. (2005). MAP kinase pathways. J. Cell Sci..

[28] Gómez-Gil E., Franco A., Vázquez-Marín B., Prieto-Ruiz F., Pérez-Díaz A., Vicente-Soler J., Madrid M., Soto T., Cansado J. (2021). Specific Functional Features of the Cell Integrity MAP Kinase Pathway in the Dimorphic Fission Yeast. J. Fungi.

[29] Kock C., Dufrêne Y.F., Heinisch J.J. (2015). Up against the wall: Is yeast cell wall integrity ensured by mechanosensing in plasma membrane microdomains?. Appl. Environ. Microbiol..

[30] Heinisch J.J., Rodicio R. (2018). Protein kinase C in fungi-more than just cell wall integrity. FEMS Microbiol. Rev..

[31] Levin D.E. (2011). Regulation of cell wall biogenesis in Saccharomyces cerevisiae: The cell wall integrity signaling pathway. Genetics.

[32] Cruz S., Muñoz S., Manjón E., García P., Sanchez Y. (2013). The fission yeast cell wall stress sensor-like proteins Mtl2 and Wsc1 act by turning on the GTPase Rho1p but act independently of the cell wall integrity pathway. Microbiologyopen.

[33] Pérez P., Cortés J.C.G., Cansado J., Ribas J.C. (2018). Fission yeast cell wall biosynthesis and cell integrity signalling. Cell Surf..

[34] Banavar S.P., Gomez C., Trogdon M., Petzold L.R., Yi T.M., Campàs O. (2018). Mechanical feedback coordinates cell wall expansion and assembly in yeast mating morphogenesis. PLoS Comput. Biol..

[35] Davì V., Tanimoto H., Ershov D., Haupt A., De Belly H., Le Borgne R., Couturier E., Boudaoud A., Minc N. (2018). Mechanosensation Dynamically Coordinates Polar Growth and Cell Wall Assembly to Promote Cell Survival. Dev. Cell.

[36] Mishra R., van Drogen F., Dechant R., Oh S., Jeon N.L., Lee S.S., Peter M. (2017). Protein kinase C and calcineurin cooperatively mediate cell survival under compressive mechanical stress. Proc. Natl. Acad. Sci. USA.

[37] Neeli-Venkata R., Diaz C.M., Celador R., Sanchez Y., Minc N. (2021). Detection of surface forces by the cell-wall mechanosensor Wsc1 in yeast. Dev. Cell.

[38] Vicente-Soler J., Soto T., Franco A., Cansado J., Madrid M. (2021). The Multiple Functions of Rho GTPases in Fission Yeasts. Cells.

[39] Hodge R.G., Ridley A.J. (2016). Regulating Rho GTPases and their regulators. Nat. Rev. Mol. Cell Biol..

[40] Pérez P., Soto T., Gómez-Gil E., Cansado J. (2020). Functional interaction between Cdc42 and the stress MAPK signaling pathway during the regulation of fission yeast polarized growth. Int. Microbiol..

[41] Arellano M., Durán A., Pérez P. (1996). Rho 1 GTPase activates the (1-3)beta-D-glucan synthase and is involved in *Schizosaccharomyces pombe* morphogenesis. EMBO J..

[42] Miller P.J., Johnson D.I. (1994). Cdc42p GTPase is involved in controlling polarized cell growth in Schizosaccharomyces pombe. Mol. Cell. Biol..

[43] Hirata D., Nakano K., Fukui M., Takenaka H., Miyakawa T., Mabuchi I. (1998). Genes that cause aberrant cell morphology by overexpression in fission yeast: A role of a small GTP-binding protein Rho2 in cell morphogenesis. J. Cell Sci..

[44] Arellano M., Duran A., Perez P. (1997). Localisation of the *Schizosaccharomyces pombe* rho1p GTPase and its involvement in the organisation of the actin cytoskeleton. J. Cell Sci..

[45] Calonge T.M., Nakano K., Arellano M., Arai R., Katayama S., Toda T., Mabuchi I., Perez P. (2000). Schizosaccharomyces pombe rho2p GTPase regulates cell wall alpha-glucan biosynthesis through the protein kinase pck2p. Mol. Biol. Cell.

[46] Sánchez-Mir L., Franco A., Martín-García R., Madrid M., Vicente-Soler J., Soto T., Gacto M., Pérez P., Cansado J. (2014). Rho2 palmitoylation is required for plasma membrane localization and proper signaling to the fission yeast cell integrity mitogen- activated protein kinase pathway. Mol. Cell. Biol..

[47] Franco A., Soto T., Martín-García R., Madrid M., Vázquez-Marín B., Vicente-Soler J., Coll P.M., Gacto M., Pérez P., Cansado J. (2017). Distinct functional relevance of dynamic GTPase cysteine methylation in fission yeast. Sci. Rep..

[48] Kabeche R., Madrid M., Cansado J., Moseley J.B. (2015). Eisosomes Regulate Phosphatidylinositol 4,5-Bisphosphate (PI(4,5)P2) Cortical Clusters and Mitogen-activated Protein (MAP) Kinase Signaling upon Osmotic Stress. J. Biol. Chem..

[49] Cansado J. (2018). To finish things well: Cysteine methylation ensures selective GTPase membrane localization and signalling. Curr. Genet..

[50] Soto T., Villar-Tajadura M.A., Madrid M., Vicente J., Gacto M., Pérez P., Cansado J. (2010). Rga4 modulates the activity of the fission yeast cell integrity MAPK pathway by acting as a Rho2 GTPase-activating protein. J. Biol. Chem..

[51] Cansado J., Soto T., Gacto M., Pérez P. (2010). Rga4, a Rho-GAP from fission yeast: Finding specificity within promiscuity. Commun. Integr. Biol..

[52] Villar-Tajadura M.A., Coll P.M., Madrid M., Cansado J., Santos B., Pérez P. (2008). Rga2 is a Rho2 GAP that regulates morphogenesis and cell integrity in S. pombe. Mol. Microbiol..

[53] Nakano K., Arai R., Mabuchi I. (1997). The small GTP-binding protein Rho1 is a multifunctional protein that regulates actin localization, cell polarity, and septum formation in the fission yeast Schizosaccharomyces pombe. Genes Cells.

[54] Arellano M., Coll P.M., Pérez P. (1999). RHO GTPases in the control of cell morphology, cell polarity, and actin localization in fission yeast. Microsc. Res. Technol..

[55] Arellano M., Valdivieso M.H., Calonge T.M., Coll P.M., Duran A., Perez P. (1999). Schizosaccharomyces pombe protein kinase C homologues, pck1p and pck2p, are targets of rho1p and rho2p and differentially regulate cell integrity. J. Cell Sci..

[56] Sayers L.G., Katayama S., Nakano K., Mellor H., Mabuchi I., Toda T., Parker P.J. (2000). Rho-dependence of *Schizosaccharomyces pombe* Pck2. Genes Cells.

[57] Sánchez-Mir L., Soto T., Franco A., Madrid M., Viana R.A., Vicente J., Gacto M., Pérez P., Cansado J. (2014). Rho1 GTPase and PKC ortholog Pck1 are upstream activators of the cell integrity MAPK pathway in fission yeast. PLoS ONE.

[58] Nakano K., Mutoh T., Mabuchi I. (2001). Characterization of GTPase-activating proteins for the function of the Rho-family small GTPases in the fission yeast Schizosaccharomyces pombe. Genes Cells.

[59] Mutoh T., Nakano K., Mabuchi I. (2005). Rho1-GEFs Rgf1 and Rgf2 are involved in formation of cell wall and septum, while Rgf3 is involved in cytokinesis in fission yeast. Genes Cells.

[60] García P., Tajadura V., García I., Sánchez Y. (2006). Role of Rho GTPases and Rho-GEFs in the regulation of cell shape and integrity in fission yeast. Yeast.

[61] Tajadura V., García B., García I., García P., Sánchez Y. (2004). Schizosaccharomyces pombe Rgf3p is a specific Rho1 GEF that regulates cell wall beta-glucan biosynthesis through the GTPase Rho1p. J. Cell Sci..

[62] Calonge T.M., Arellano M., Coll P.M., Perez P. (2003). Rga5p is a specific Rho1p GTPase-activating protein that regulates cell integrity in Schizosaccharomyces pombe. Mol. Microbiol..

[63] García P., Tajadura V., García I., Sánchez Y. (2006). Rgf1p is a specific Rho1-GEF that coordinates cell polarization with cell wall biogenesis in fission yeast. Mol. Biol. Cell.

[64] Barba G., Soto T., Madrid M., Núñez A., Vicente J., Gacto M., Cansado J., Group Y.P. (2008). Activation of the cell integrity pathway is channelled through diverse signalling elements in fission yeast. Cell. Signal..

[65] Nakano K., Mutoh T., Arai R., Mabuchi I. (2003). The small GTPase Rho4 is involved in controlling cell morphology and septation in fission yeast. Genes Cells.

[66] Santos B., Martín-Cuadrado A.B., Vázquez de Aldana C.R., del Rey F., Pérez P. (2005). Rho4 GTPase is involved in secretion of glucanases during fission yeast cytokinesis. Eukaryot. Cell.

[67] Santos B., Gutiérrez J., Calonge T.M., Pérez P. (2003). Novel Rho GTPase involved in cytokinesis and cell wall integrity in the fission yeast Schizosaccharomyces pombe. Eukaryot. Cell.

[68] Nakano K., Arai R., Mabuchi I. (2005). Small GTPase Rho5 is a functional homologue of Rho1, which controls cell shape and septation in fission yeast. FEBS Lett..

[69] Rincón S.A., Santos B., Pérez P. (2006). Fission yeast Rho5p GTPase is a functional paralogue of Rho1p that plays a role in survival of spores and stationary-phase cells. Eukaryot. Cell.

[70] Doi A., Kita A., Kanda Y., Uno T., Asami K., Satoh R., Nakano K., Sugiura R. (2015). Geranylgeranyltransferase Cwg2-Rho4/Rho5 module is implicated in the Pmk1 MAP kinase-mediated cell wall integrity pathway in fission yeast. Genes Cells.

[71] Newton A.C. (2018). Protein kinase C: Perfectly balanced. Crit. Rev. Biochem. Mol. Biol..

[72] Madrid M., Vazquez-Marin B., Franco A., Soto T., Vicente-Soler J., Gacto M., Cansado J. (2016). Multiple crosstalk between TOR and the cell integrity MAPK signaling pathway in fission yeast. Sci. Rep..

[73] Freeley M., Kelleher D., Long A. (2011). Regulation of Protein Kinase C function by phosphorylation on conserved and non-conserved sites. Cell. Signal..

[74] Madrid M., Vázquez-Marín B., Soto T., Franco A., Gómez-Gil E., Vicente-Soler J., Gacto M., Pérez P., Cansado J. (2017). Differential functional regulation of protein kinase C (PKC) orthologs in fission yeast. J. Biol. Chem..

[75] Toda T., Shimanuki M., Yanagida M. (1993). Two novel protein kinase C-related genes of fission yeast are essential for cell viability and implicated in cell shape control. EMBO J..

[76] Matsuyama A., Arai R., Yashiroda Y., Shirai A., Kamata A., Sekido S., Kobayashi Y., Hashimoto A., Hamamoto M., Hiraoka Y. (2006). ORFeome cloning and global analysis of protein localization in the fission yeast Schizosaccharomyces pombe. Nat. Biotechnol..

[77] Magliozzi J.O., Sears J., Cressey L., Brady M., Opalko H.E., Kettenbach A.N., Moseley J.B. (2020). Fission yeast Pak1 phosphorylates anillin-like Mid1 for spatial control of cytokinesis. J. Cell Biol..

[78] Madrid M., Jiménez R., Sánchez-Mir L., Soto T., Franco A., Vicente-Soler J., Gacto M., Pérez P., Cansado J. (2015). Multiple layers of regulation influence cell integrity control by the PKC ortholog Pck2 in fission yeast. J. Cell Sci..

[79] Kanda Y., Satoh R., Takasaki T., Tomimoto N., Tsuchiya K., Tsai C.A., Tanaka T., Kyomoto S., Hamada K., Fujiwara T. (2021). Sequestration of the PKC ortholog Pck2 in stress granules as a feedback mechanism of MAPK signaling in fission yeast. J. Cell Sci..

[80] Newton A.C. (2010). Protein kinase C: Poised to signal. Am. J. Physiol. Endocrinol. Metab..

[81] Niederberger C., Schweingruber M.E. (1999). A *Schizosaccharomyces pombe* gene, ksg1, that shows structural homology to the human phosphoinositide-dependent protein kinase PDK1, is essential for growth, mating and sporulation. Mol. Gen. Genet..

[82] Gräub R., Hilti N., Niederberger C., Schweingruber M.E. (2003). Ksg1, a homologue of the phosphoinositide-dependent protein kinase 1, controls cell wall integrity in Schizosaccharomyces pombe. J. Basic Microbiol..

[83] Madrid M., Fernández-Zapata J., Sánchez-Mir L., Soto T., Franco A., Vicente-Soler J., Gacto M., Cansado J. (2013). Role of the fission yeast cell integrity MAPK pathway in response to glucose limitation. BMC Microbiol..

[84] Kanda Y., Satoh R., Matsumoto S., Ikeda C., Inutsuka N., Hagihara K., Matzno S., Tsujimoto S., Kita A., Sugiura R. (2016). Skb5, an SH3 adaptor protein, regulates Pmk1 MAPK signaling by controlling the intracellular localization of the MAPKKK Mkh1. J. Cell Sci..

[85] Yang P., Pimental R., Lai H., Marcus S. (1999). Direct activation of the fission yeast PAK Shk1 by the novel SH3 domain protein, Skb5. J. Biol. Chem..

[86] Vázquez B., Soto T., del Dedo J.E., Franco A., Vicente J., Hidalgo E., Gacto M., Cansado J., Madrid M. (2015). Distinct biological activity of threonine monophosphorylated MAPK isoforms during the stress response in fission yeast. Cell. Signal..

[87] Sánchez-Mir L., Franco A., Madrid M., Vicente-Soler J., Villar-Tajadura M.A., Soto T., Pérez P., Gacto M., Cansado J. (2012). Biological significance of nuclear localization of mitogen-activated protein kinase Pmk1 in fission yeast. J. Biol. Chem..

[88] Soto T., Núñez A., Madrid M., Vicente J., Gacto M., Cansado J. (2007). Transduction of centrifugation-induced gravity forces through mitogen-activated protein kinase pathways in the fission yeast Schizosaccharomyces pombe. Microbiology.

[89] Kaino T., Tonoko K., Mochizuki S., Takashima Y., Kawamukai M. (2018). Schizosaccharomyces japonicus has low levels of CoQ(10) synthesis, respiration deficiency, and efficient ethanol production. Biosci. Biotechnol. Biochem..

[90] Degols G., Shiozaki K., Russell P. (1996). Activation and regulation of the Spc1 stress-activated protein kinase in Schizosaccharomyces pombe. Mol. Cell. Biol..

[91] Shiozaki K., Russell P. (1996). Conjugation, meiosis, and the osmotic stress response are regulated by Spc1 kinase through Atf1 transcription factor in fission yeast. Genes Dev..

[92] Wilkinson M.G., Samuels M., Takeda T., Toone W.M., Shieh J.C., Toda T., Millar J.B., Jones N. (1996). The Atf1 transcription factor is a target for the Sty1 stress-activated MAP kinase pathway in fission yeast. Genes Dev..

[93] Lawrence C.L., Maekawa H., Worthington J.L., Reiter W., Wilkinson C.R., Jones N. (2007). Regulation of *Schizosaccharomyces pombe* Atf1 protein levels by Sty1-mediated phosphorylation and heterodimerization with Pcr1. J. Biol. Chem..

[94] Chen D., Wilkinson C.R., Watt S., Penkett C.J., Toone W.M., Jones N., Bähler J. (2008). Multiple pathways differentially regulate global oxidative stress responses in fission yeast. Mol. Biol. Cell.

[95] Reiter W., Watt S., Dawson K., Lawrence C.L., Bahler J., Jones N., Wilkinson C.R. (2008). Fission yeast MAP kinase Sty1 is recruited to stress-induced genes. J. Biol. Chem..

[96] Takada H., Nishimura M., Asayama Y., Mannse Y., Ishiwata S., Kita A., Doi A., Nishida A., Kai N., Moriuchi S. (2007). Atf1 is a target of the mitogen-activated protein kinase Pmk1 and regulates cell integrity in fission yeast. Mol. Biol. Cell.

[97] Takada H., Nishida A., Domae M., Kita A., Yamano Y., Uchida A., Ishiwata S., Fang Y., Zhou X., Masuko T. (2010). The cell surface protein gene ecm33+ is a target of the two transcription factors Atf1 and Mbx1 and negatively regulates Pmk1 MAPK cell integrity signaling in fission yeast. Mol. Biol. Cell.

[98] Papadopoulou K., Ng S.S., Ohkura H., Geymonat M., Sedgwick S.G., McInerny C.J. (2008). Regulation of gene expression during M-G1-phase in fission yeast through Plo1p and forkhead transcription factors. J. Cell Sci..

[99] Oliveira C., Faoro H., Alves L.R., Goldenberg S. (2017). RNA-binding proteins and their role in the regulation of gene expression in Trypanosoma cruzi and Saccharomyces cerevisiae. Genet. Mol. Biol..

[100] Sugiura R., Satoh R., Ishiwata S., Umeda N., Kita A. (2011). Role of RNA-Binding Proteins in MAPK Signal Transduction Pathway. J. Signal Transduct..

[101] Venigalla R.K., Turner M. (2012). RNA-binding proteins as a point of convergence of the PI3K and p38 MAPK pathways. Front. Immunol..

[102] Hasan A., Cotobal C., Duncan C.D., Mata J. (2014). Systematic analysis of the role of RNA-binding proteins in the regulation of RNA stability. PLoS Genet..

[103] Díaz-Cuervo H., Bueno A. (2008). Cds1 controls the release of Cdc14-like phosphatase Flp1 from the nucleolus to drive full activation of the checkpoint response to replication stress in fission yeast. Mol. Biol. Cell.

[104] Broadus M.R., Gould K.L. (2012). Multiple protein kinases influence the redistribution of fission yeast Clp1/Cdc14 phosphatase upon genotoxic stress. Mol. Biol. Cell.

[105] Sugiura R., Kita A., Shimizu Y., Shuntoh H., Sio S.O., Kuno T. (2003). Feedback regulation of MAPK signalling by an RNA-binding protein. Nature.

[106] Tsukahara K., Yamamoto H., Okayama H. (1998). An RNA binding protein negatively controlling differentiation in fission yeast. Mol. Cell. Biol..

[107] Jeong H.T., Ozoe F., Tanaka K., Nakagawa T., Matsuda H., Kawamukai M. (2004). A novel gene, msa1, inhibits sexual differentiation in Schizosaccharomyces pombe. Genetics.

[108] Jeong H.T., Oowatari Y., Abe M., Tanaka K., Matsuda H., Kawamukai M. (2004). Interaction between a negative regulator (Msa2/Nrd1) and a positive regulator (Cpc2) of sexual differentiation in Schizosaccharomyces pombe. Biosci. Biotechnol. Biochem..

[109] Mata J., Bähler J. (2006). Global roles of Ste11p, cell type, and pheromone in the control of gene expression during early sexual differentiation in fission yeast. Proc. Natl. Acad. Sci. USA.

[110] Satoh R., Morita T., Takada H., Kita A., Ishiwata S., Doi A., Hagihara K., Taga A., Matsumura Y., Tohda H. (2009). Role of the RNA-binding Protein Nrd1 and Pmk1 Mitogen-activated Protein Kinase in the Regulation of Myosin mRNA Stability in Fission Yeast. Mol. Biol. Cell.

[111] Pollard T.D., Wu J.Q. (2010). Understanding cytokinesis: Lessons from fission yeast. Nat. Rev. Mol. Cell Biol..

[112] Kobayashi A., Kanaba T., Satoh R., Fujiwara T., Ito Y., Sugiura R., Mishima M. (2013). Structure of the second RRM domain of Nrd1, a fission yeast MAPK target RNA binding protein, and implication for its RNA recognition and regulation. Biochem. Biophys. Res. Commun..

[113] Satoh R., Tanaka A., Kita A., Morita T., Matsumura Y., Umeda N., Takada M., Hayashi S., Tani T., Shinmyozu K. (2012). Role of the RNA-Binding Protein Nrd1 in Stress Granule Formation and Its Implication in the Stress Response in Fission Yeast. PLoS ONE.

[114] Oowatari Y., Jeong H., Tanae K., Nakagawa T., Kawamukai M. (2011). Regulation and role of an RNA-binding protein Msa2 in controlling the sexual differentiation of fission yeast. Curr. Genet..

[115] Kettenbach A.N., Deng L., Wu Y., Baldissard S., Adamo M.E., Gerber S.A., Moseley J.B. (2015). Quantitative phosphoproteomics reveals pathways for coordination of cell growth and division by the conserved fission yeast kinase pom1. Mol. Cell. Proteomics.

[116] Swaffer M.P., Jones A.W., Flynn H.R., Snijders A.P., Nurse P. (2018). Quantitative Phosphoproteomics Reveals the Signaling Dynamics of Cell-Cycle Kinases in the Fission Yeast Schizosaccharomyces pombe. Cell Rep..

[117] Tay Y.D., Leda M., Spanos C., Rappsilber J., Goryachev A.B., Sawin K.E. (2019). Fission Yeast NDR/LATS Kinase Orb6 Regulates Exocytosis via Phosphorylation of the Exocyst Complex. Cell Rep..

[118] Nunez A., Franco A., Madrid M., Soto T., Vicente J., Gacto M., Cansado J. (2009). Role for RACK1 Orthologue Cpc2 in the Modulation of Stress Response in Fission Yeast. Mol. Biol. Cell.

[119] Hollingworth D., Candel A.M., Nicastro G., Martin S.R., Briata P., Gherzi R., Ramos A. (2012). KH domains with impaired nucleic acid binding as a tool for functional analysis. Nucleic Acids Res..

[120] Grishin N.V. (2001). KH domain: One motif, two folds. Nucleic Acids Res..

[121] Sugiura R., Kita A., Kuno T. (2004). Upregulation of mRNA in MAPK signaling—Transcriptional activation or mRNA stabilization?. Cell Cycle.

[122] Satoh R., Matsumura Y., Tanaka A., Takada M., Ito Y., Hagihara K., Inari M., Kita A., Fukao A., Fujiwara T. (2017). Spatial regulation of the KH domain RNA-binding protein Rnc1 mediated by a Crm1-independent nuclear export system in Schizosaccharomyces pombe. Mol. Microbiol..

[123] Satoh R., Hagihara K., Sugiura R. (2018). Rae1-mediated nuclear export of Rnc1 is an important determinant in controlling MAPK signaling. Curr. Genet..

[124] Satoh R., Hara N., Kawasaki A., Takasaki T., Sugiura R. (2018). Distinct modes of stress granule assembly mediated by the KH-type RNA-binding protein Rnc1. Genes Cells.

[125] Prieto-Ruiz F., Vicente-Soler J., Franco A., Gomez-Gil E., Sanchez-Marinas M., Vazquez-Marin B., Aligue R., Madrid M., Moreno S., Soto T. (2020). RNA-Binding Protein Rnc1 Regulates Cell Length at Division and Acute Stress Response in Fission Yeast through Negative Feedback Modulation of the Stress-Activated Mitogen-Activated Protein Kinase Pathway. mBio.

[126] Hirayama S., Sugiura R., Lu Y., Maeda T., Kawagishi K., Yokoyama M., Tohda H., Giga-Hama Y., Shuntoh H., Kuno T. (2003). Zinc finger protein Prz1 regulates Ca^2+^ but not Cl^−^ homeostasis in fission yeast. Identification of distinct branches of calcineurin signaling pathway in fission yeast. J. Biol. Chem..

[127] Deng L., Sugiura R., Takeuchi M., Suzuki M., Ebina H., Takami T., Koike A., Iba S., Kuno T. (2006). Real-time monitoring of calcineurin activity in living cells: Evidence for two distinct Ca^2+^-dependent pathways in fission yeast. Mol. Biol. Cell.

[128] Hamasaki-Katagiri N., Ames J.B. (2010). Neuronal calcium sensor-1 (Ncs1p) is up-regulated by calcineurin to promote Ca2+ tolerance in fission yeast. J. Biol. Chem..

[129] Maeda T., Sugiura R., Kita A., Saito M., Deng L., He Y., Yabin L., Fujita Y., Takegawa K., Shuntoh H. (2004). Pmr1, a P-type ATPase, and Pdt1, an Nramp homologue, cooperatively regulate cell morphogenesis in fission yeast: The importance of Mn2+ homeostasis. Genes Cells.

[130] Cisneros-Barroso E., Yance-Chávez T., Kito A., Sugiura R., Gómez-Hierro A., Giménez-Zaragoza D., Aligue R. (2014). Negative feedback regulation of calcineurin-dependent Prz1 transcription factor by the CaMKK-CaMK1 axis in fission yeast. Nucleic Acids Res..

[131] Chatfield-Reed K., Vachon L., Kwon E.J., Chua G. (2016). Conserved and Diverged Functions of the Calcineurin-Activated Prz1 Transcription Factor in Fission Yeast. Genetics.

[132] Yoshida T., Toda T., Yanagida M. (1994). A calcineurin-like gene ppb1+ in fission yeast: Mutant defects in cytokinesis, cell polarity, mating and spindle pole body positioning. J. Cell Sci..

[133] Aydar E., Palmer C.P. (2009). Polycystic kidney disease channel and synaptotagmin homologues play roles in schizosaccharomyces pombe cell wall synthesis/repair and membrane protein trafficking. J. Membr. Biol..

[134] Carnero E., Ribas J.C., García B., Durán A., Sánchez Y. (2000). *Schizosaccharomyces pombe* ehs1p is involved in maintaining cell wall integrity and in calcium uptake. Mol. Gen. Genet..

[135] Ma Y., Sugiura R., Koike A., Ebina H., Sio S.O., Kuno T. (2011). Transient receptor potential (TRP) and Cch1-Yam8 channels play key roles in the regulation of cytoplasmic Ca^2+^ in fission yeast. PLoS ONE.

[136] Gow N.A.R., Latge J.P., Munro C.A. (2017). The Fungal Cell Wall: Structure, Biosynthesis, and Function. Microbiol. Spectr..

[137] Hopke A., Brown A.J.P., Hall R.A., Wheeler R.T. (2018). Dynamic Fungal Cell Wall Architecture in Stress Adaptation and Immune Evasion. Trends Microbiol..

[138] Mazur P., Baginsky W. (1996). In vitro activity of 1,3-beta-D-glucan synthase requires the GTP-binding protein Rho1. J. Biol. Chem..

[139] Guo S., Shen X., Yan G., Ma D., Bai X., Li S., Jiang Y. (2009). A MAP Kinase Dependent Feedback Mechanism Controls Rho1 GTPase and Actin Distribution in Yeast. PLoS ONE.

[140] Kampmeyer C., Johansen J.V., Holmberg C., Karlson M., Gersing S.K., Bordallo H.N., Kragelund B.B., Lerche M.H., Jourdain I., Winther J.R. (2020). Mutations in a Single Signaling Pathway Allow Cell Growth in Heavy Water. ACS Synth. Biol..

[141] Imai Y., Shimasaki T., Enokimura C., Ohtsuka H., Tsubouchi S., Ihara K., Aiba H. (2020). gas1 mutation extends chronological lifespan via Pmk1 and Sty1 MAPKs in Schizosaccharomyces pombe. Biosci. Biotechnol. Biochem..

[142] Merla A., Johnson D.I. (2001). The *Schizosaccharomyces pombe* Cdc42p GTPase signals through Pak2p and the Mkh1p-Pek1p-Spm1p MAP kinase pathway. Curr. Genet..

[143] Kono K., Saeki Y., Yoshida S., Tanaka K., Pellman D. (2012). Proteasomal degradation resolves competition between cell polarization and cellular wound healing. Cell.

[144] Gu Y., Oliferenko S. (2015). Comparative biology of cell division in the fission yeast clade. Curr. Opin. Microbiol..

[145] Rincon S.A., Paoletti A. (2016). Molecular control of fission yeast cytokinesis. Semin. Cell Dev. Biol..

[146] Garcia Cortes J.C., Ramos M., Osumi M., Perez P., Ribas J.C. (2016). The Cell Biology of Fission Yeast Septation. Microbiol. Mol. Biol. Rev..

[147] Tolliday N., VerPlank L., Li R. (2002). Rho1 directs formin-mediated actin ring assembly during budding yeast cytokinesis. Curr. Biol..

[148] Chin C.F., Bennett A.M., Ma W.K., Hall M.C., Yeong F.M. (2012). Dependence of Chs2 ER export on dephosphorylation by cytoplasmic Cdc14 ensures that septum formation follows mitosis. Mol. Biol. Cell.

[149] Yoshida S., Bartolini S., Pellman D. (2009). Mechanisms for concentrating Rho1 during cytokinesis. Genes Dev..

[150] García R., Bermejo C., Grau C., Pérez R., Rodríguez-Peña J.M., Francois J., Nombela C., Arroyo J. (2004). The global transcriptional response to transient cell wall damage in *Saccharomyces cerevisiae* and its regulation by the cell integrity signaling pathway. J. Biol. Chem..

[151] Cabib E., Arroyo J. (2013). How carbohydrates sculpt cells: Chemical control of morphogenesis in the yeast cell wall. Nat. Rev. Microbiol..

[152] Piña F.J., Niwa M. (2015). The ER Stress Surveillance (ERSU) pathway regulates daughter cell ER protein aggregate inheritance. Elife.

[153] Babour A., Bicknell A.A., Tourtellotte J., Niwa M. (2010). A surveillance pathway monitors the fitness of the endoplasmic reticulum to control its inheritance. Cell.

[154] Edreira T., Celador R., Manjón E., Sánchez Y. (2020). A novel checkpoint pathway controls actomyosin ring constriction trigger in fission yeast. eLife.

[155] Jin Q.-W., Zhou M., Bimbo A., Balasubramanian M.K., McCollum D. (2006). A role for the septation initiation network in septum assembly revealed by genetic analysis of sid2-250 suppressors. Genetics.

[156] Davidson R., Laporte D., Wu J.-Q. (2014). Regulation of Rho-GEF Rgf3 by the arrestin Art1 in fission yeast cytokinesis. Mol. Biol. Cell.

[157] Martín-García R., Arribas V., Coll P.M., Pinar M., Viana R.A., Rincón S.A., Correa-Bordes J., Ribas J.C., Pérez P. (2018). Paxillin-Mediated Recruitment of Calcineurin to the Contractile Ring Is Required for the Correct Progression of Cytokinesis in Fission Yeast. Cell Rep..

[158] Mascaraque V., Hernáez M.L., Jiménez-Sánchez M., Hansen R., Gil C., Martín H., Cid V.J., Molina M. (2013). Phosphoproteomic analysis of protein kinase C signaling in *Saccharomyces cerevisiae* reveals Slt2 mitogen-activated protein kinase (MAPK)-dependent phosphorylation of eisosome core components. Mol. Cell Proteom..

[159] Dodgson J., Chessel A., Vaggi F., Giordan M., Yamamoto M., Arai K., Madrid M., Geymonat M., Abenza J.F., Cansado J. (2017). Reconstructing regulatory pathways by systematically mapping protein localization interdependency networks. bioRxiv.

[160] Cortés J.C., Konomi M., Martins I.M., Muñoz J., Moreno M.B., Osumi M., Durán A., Ribas J.C. (2007). The (1,3)beta-D-glucan synthase subunit Bgs1p is responsible for the fission yeast primary septum formation. Mol. Microbiol..

[161] Gotoh Y., Nishida E., Shimanuki M., Toda T., Imai Y., Yamamoto M. (1993). Schizosaccharomyces pombe Spk1 is a tyrosine-phosphorylated protein functionally related to Xenopus mitogen-activated protein kinase. Mol. Cell. Biol..

[162] Millar J.B., Buck V., Wilkinson M.G. (1995). Pyp1 and Pyp2 PTPases dephosphorylate an osmosensing MAP kinase controlling cell size at division in fission yeast. Genes Dev..

[163] Shiozaki K., Russell P. (1995). Counteractive roles of protein phosphatase 2C (PP2C) and a MAP kinase kinase homolog in the osmoregulation of fission yeast. EMBO J..

[164] Didmon M., Davis K., Watson P., Ladds G., Broad P., Davey J. (2002). Identifying regulators of pheromone signalling in the fission yeast Schizosaccharomyces pombe. Curr. Genet..

[165] Degols G., Russell P. (1997). Discrete roles of the Spc1 kinase and the Atf1 transcription factor in the UV response of Schizosaccharomyces pombe. Mol. Cell. Biol..

[166] Soto T., Beltrán F.F., Paredes V., Madrid M., Millar J.B., Vicente-Soler J., Cansado J., Gacto M. (2002). Cold induces stress-activated protein kinase-mediated response in the fission yeast Schizosaccharomyces pombe. Eur. J. Biochem..

[167] Quinn J., Findlay V.J., Dawson K., Millar J.B., Jones N., Morgan B.A., Toone W.M. (2002). Distinct regulatory proteins control the graded transcriptional response to increasing H(2)O(2) levels in fission yeast Schizosaccharomyces pombe. Mol. Biol. Cell.

[168] Rodríguez-Gabriel M.A., Russell P. (2005). Distinct signaling pathways respond to arsenite and reactive oxygen species in Schizosaccharomyces pombe. Eukaryot. Cell.

[169] George V.T., Brooks G., Humphrey T.C. (2007). Regulation of cell cycle and stress responses to hydrostatic pressure in fission yeast. Mol. Biol. Cell.

[170] Buck V., Quinn J., Soto Pino T., Martin H., Saldanha J., Makino K., Morgan B.A., Millar J.B. (2001). Peroxide sensors for the fission yeast stress-activated mitogen-activated protein kinase pathway. Mol. Biol. Cell.

[171] Hohmann S. (2002). Osmotic stress signaling and osmoadaptation in yeasts. Microbiol. Mol. Biol. Rev. MMBR.

[172] Samejima I., Mackie S., Fantes P.A. (1997). Multiple modes of activation of the stress-responsive MAP kinase pathway in fission yeast. EMBO J..

[173] Nguyen A.N., Shiozaki K. (1999). Heat-shock-induced activation of stress MAP kinase is regulated by threonine- and tyrosine-specific phosphatases. Genes Dev..

[174] Gaits F., Russell P. (1999). Active nucleocytoplasmic shuttling required for function and regulation of stress-activated kinase Spc1/StyI in fission yeast. Mol. Biol. Cell.

[175] Doi K., Gartner A., Ammerer G., Errede B., Shinkawa H., Sugimoto K., Matsumoto K. (1994). MSG5, a novel protein phosphatase promotes adaptation to pheromone response in S. cerevisiae. EMBO J..

[176] Watanabe Y., Irie K., Matsumoto K. (1995). Yeast RLM1 encodes a serum response factor-like protein that may function downstream of the Mpk1 (Slt2) mitogen-activated protein kinase pathway. Mol. Cell. Biol..

[177] Davenport K.D., Williams K.E., Ullmann B.D., Gustin M.C. (1999). Activation of the *Saccharomyces cerevisiae* filamentation/invasion pathway by osmotic stress in high-osmolarity glycogen pathway mutants. Genetics.

[178] Martín H., Rodríguez-Pachón J.M., Ruiz C., Nombela C., Molina M. (2000). Regulatory mechanisms for modulation of signaling through the cell integrity Slt2-mediated pathway in Saccharomyces cerevisiae. J. Biol. Chem..

[179] González A., Hall M.N. (2017). Nutrient sensing and TOR signaling in yeast and mammals. EMBO J..

[180] Wullschleger S., Loewith R., Hall M.N. (2006). TOR signaling in growth and metabolism. Cell.

[181] Cybulski N., Hall M.N. (2009). TOR complex 2: A signaling pathway of its own. Trends Biochem. Sci..

[182] Loewith R. (2011). A brief history of TOR. Biochem. Soc. Trans..

[183] Alvarez B., Moreno S. (2006). Fission yeast Tor2 promotes cell growth and represses cell differentiation. J. Cell Sci..

[184] Nakashima A., Otsubo Y., Yamashita A., Sato T., Yamamoto M., Tamanoi F. (2012). Psk1, an AGC kinase family member in fission yeast, is directly phosphorylated and controlled by TORC1 and functions as S6 kinase. J. Cell Sci..

[185] Valbuena N., Rozalén A.E., Moreno S. (2012). Fission yeast TORC1 prevents eIF2α phosphorylation in response to nitrogen and amino acids via Gcn2 kinase. J. Cell Sci..

[186] Weisman R., Choder M. (2001). The fission yeast TOR homolog, tor1+, is required for the response to starvation and other stresses via a conserved serine. J. Biol. Chem..

[187] Matsuo T., Kubo Y., Watanabe Y., Yamamoto M. (2003). Schizosaccharomyces pombe AGC family kinase Gad8p forms a conserved signaling module with TOR and PDK1-like kinases. EMBO J..

[188] Ikeda K., Morigasaki S., Tatebe H., Tamanoi F., Shiozaki K. (2008). Fission yeast TOR complex 2 activates the AGC-family Gad8 kinase essential for stress resistance and cell cycle control. Cell Cycle.

[189] Duran R.V., Hall M.N. (2012). Regulation of TOR by small GTPases. EMBO Rep..

[190] Mendoza M.C., Er E.E., Blenis J. (2011). The Ras-ERK and PI3K-mTOR pathways: Cross-talk and compensation. Trends Biochem. Sci..

[191] Morigasaki S., Chin L.C., Hatano T., Emori M., Iwamoto M., Tatebe H., Shiozaki K. (2019). Modulation of TOR complex 2 signaling by the stress-activated MAPK pathway in fission yeast. J. Cell Sci..

[192] Petersen J., Hagan I.M. (2005). Polo kinase links the stress pathway to cell cycle control and tip growth in fission yeast. Nature.

[193] Torres J., Di Como C.J., Herrero E., De La Torre-Ruiz M.A. (2002). Regulation of the cell integrity pathway by rapamycin-sensitive TOR function in budding yeast. J. Biol. Chem..

[194] Hatano T., Morigasaki S., Tatebe H., Ikeda K., Shiozaki K. (2015). Fission yeast Ryh1 GTPase activates TOR Complex 2 in response to glucose. Cell Cycle.

[195] Zhan K., Narasimhan J., Wek R.C. (2004). Differential activation of eIF2 kinases in response to cellular stresses in Schizosaccharomyces pombe. Genetics.

[196] Berlanga J.J., Rivero D., Martín R., Herrero S., Moreno S., de Haro C. (2010). Role of mitogen-activated protein kinase Sty1 in regulation of eukaryotic initiation factor 2alpha kinases in response to environmental stress in Schizosaccharomyces pombe. Eukaryot. Cell.

[197] Asano K. (2021). Origin of translational control by eIF2α phosphorylation: Insights from genome-wide translational profiling studies in fission yeast. Curr. Genet..

[198] Nakashima A., Sato T., Tamanoi F. (2010). Fission yeast TORC1 regulates phosphorylation of ribosomal S6 proteins in response to nutrients and its activity is inhibited by rapamycin. J. Cell Sci..

[199] Du W., Hálová L., Kirkham S., Atkin J., Petersen J. (2012). TORC2 and the AGC kinase Gad8 regulate phosphorylation of the ribosomal protein S6 in fission yeast. Biol. Open.

[200] Omnus D.J., Manford A.G., Bader J.M., Emr S.D., Stefan C.J. (2016). Phosphoinositide kinase signaling controls ER-PM cross-talk. Mol. Biol. Cell.

[201] Du W., Forte G.M., Smith D., Petersen J. (2016). Phosphorylation of the amino-terminus of the AGC kinase Gad8 prevents its interaction with TORC2. Open Biol..

[202] Tatebe H., Murayama S., Yonekura T., Hatano T., Richter D., Furuya T., Kataoka S., Furuita K., Kojima C., Shiozaki K. (2017). Substrate specificity of TOR complex 2 is determined by a ubiquitin-fold domain of the Sin1 subunit. eLife.

[203] García R., Bravo E., Diez-Muñiz S., Nombela C., Rodríguez-Peña J.M., Arroyo J. (2017). A novel connection between the Cell Wall Integrity and the PKA pathways regulates cell wall stress response in yeast. Sci. Rep..

[204] Gerik K.J., Donlin M.J., Soto C.E., Banks A.M., Banks I.R., Maligie M.A., Selitrennikoff C.P., Lodge J.K. (2005). Cell wall integrity is dependent on the PKC1 signal transduction pathway in Cryptococcus neoformans. Mol. Microbiol..

[205] Donlin M.J., Upadhya R., Gerik K.J., Lam W., VanArendonk L.G., Specht C.A., Sharma N.K., Lodge J.K. (2014). Cross talk between the cell wall integrity and cyclic AMP/protein kinase A pathways in Cryptococcus neoformans. mBio.

[206] Higuchi T., Watanabe Y., Yamamoto M. (2002). Protein kinase A regulates sexual development and gluconeogenesis through phosphorylation of the Zn finger transcriptional activator Rst2p in fission yeast. Mol. Cell. Biol..

[207] Hoffman C.S. (2005). Glucose sensing via the protein kinase A pathway in Schizosaccharomyces pombe. Biochem. Soc. Trans..

[208] Hoffman C.S. (2005). Except in every detail: Comparing and contrasting G-protein signaling in *Saccharomyces cerevisiae* and *Schizosaccharomyces pombe*. Eukaryot. Cell.

